# Socioeconomic Disadvantage and Youth Mental Health During the COVID-19 Pandemic Lockdown

**DOI:** 10.1001/jamanetworkopen.2024.20466

**Published:** 2024-07-05

**Authors:** Shana Adise, Amy E. West, Panteha Hayati Rezvan, Andrew T. Marshall, Samantha Betts, Eric Kan, Elizabeth R. Sowell

**Affiliations:** 1 Department of Pediatrics, Division of Endocrinology, Diabetes and Metabolism, Children’s Hospital Los Angeles, Los Angeles, California; 2 Department of Pediatrics, Division of General Pediatrics, Children’s Hospital Los Angeles, Los Angeles, California; 3 Biostatistics and Data Management Core, The Saban Research Institute, Children’s Hospital of Los Angeles, Los Angeles, California; 4 Department of Pediatrics, Division of Pediatric Research Administration, Children’s Hospital of Los Angeles, Los Angeles, California; 5 Department of Pediatrics, Division of Neurology, Children’s Hospital Los Angeles, Los Angeles, California

## Abstract

**Importance:**

Adolescence is a period in which mental health problems emerge. Research suggests that the COVID-19 lockdown may have worsened emotional and behavioral health.

**Objective:**

To examine whether socioeconomic status was associated with mental health outcomes among youths during the COVID-19 pandemic.

**Design, Setting, and Participants:**

The Adolescent Brain Cognitive Development (ABCD) Study is a multisite 10-year longitudinal study of youth neurocognitive development in the US. Recruitment was staggered where the baseline visit (ages 9 to 10 years) occurred from 2016 to 2018, and visits occurred yearly. The COVID-19 lockdown halted research collection during the 2-year follow-up visits (ages 11 to 12 years), but eventually resumed. As some youths already underwent their 2-year visits prior to lockdown, this allowed for a natural experiment-like design to compare prepandemic and intrapandemic groups. Thus, data were gathered from the 1-year follow-up (pre–COVID-19 lockdown for all youths) and the 2-year follow-up, of which a portion of youths had data collected after the lockdown began, to compare whether a period of near social isolation was associated with mental health symptoms in youths. The prepandemic group consisted of youths with a 2-year follow-up visit collected prior to March 11, 2020, and the intrapandemic group had their 2-year follow-up visit after lockdown restrictions were lifted.

**Main Outcomes and Measures:**

Assessments included measures on income-to-needs ratio (INR; derived from total household income), the Child Behavior Checklist (a measure of mental health symptomology), and the Family Environmental Scale.

**Results:**

The final sample included 10 399 youths; 3947 (52.3%) were male; 2084 (20.3%) were Latinx/Hispanic; 6765 (66.0%) were White; 4600 (44.2%) reported caregiver education levels below a 4-year college degree; and 2475 (26.2%) had INR either below 100% (indicating poverty) or between 100% and less than 200% (near poverty). Among youths in the intrapandemic group, worse mental health symptoms (eg, more total problems, greater depression, and greater anxiety) over time were associated with being from a household with higher socioeconomic status (eg, when comparing individuals who differed by 1 unit on INR between prepandemic and intrapandemic groups from 1-year to 2-year follow-up, their expected difference in total problems score was 0.79 [95% CI, 0.37-1.22]; false discovery rate–corrected *P* < .001).

**Conclusions and Relevance:**

This cohort study found that the COVID-19 lockdown was associated with disproportionately negative mental health outcomes among youths from higher socioeconomic status backgrounds. Although this study does not shed light on the direct mechanisms driving these associations, it does provide some support for positive outcomes for youths. Future studies are needed to understand whether these associations persist over longer periods of time.

## Key Points

**Question:**

Did the COVID-19 lockdown disproportionately affect mental health symptoms among youths in lower income households?

**Findings:**

In this cohort study of 10 399 youths, the COVID-19 lockdown was associated with worsening mental health symptoms among youths in higher-income households.

**Meaning:**

These results suggest that socioeconomic status may matter when considering the youth mental health outcomes of the COVID-19 lockdown, which may be important for targeted treatment approaches.

## Teaser

This cohort study examines whether household socioeconomic status is associated with mental health outcomes among youths during the COVID-19 pandemic.

## Introduction

To stop the spread of the COVID-19 virus, a months-long, countrywide lockdown limiting social interactions was set in place. Although presumed beneficial, for youths, the COVID-19 pandemic and its associated lockdown has had profound effects, including negative effects on mental health.^1^ The combination of infection threat and social isolation increased fear, helplessness, insomnia, and boredom, while isolation heightened risk for depression, anxiety, and posttraumatic stress^2^; these emotional and behavioral problems were partially influenced by family factors and parental distress.^3,4^ This presumed increase in emotional and behavioral problems for adolescents is particularly concerning. Adolescence is a developmental time of risk for the emergence of mental health problems, which are exacerbated among youths from low socioeconomic status households.^5^ Unfortunately, the pandemic negatively (and disproportionately) affected individuals from lower income families.^6^ As such, disruption to daily activities may have exacerbated mental health symptoms disproportionately among those who live in low socioeconomic status households. However, to our knowledge, no studies have elucidated how the COVID-19 lockdown period may have exacerbated mental health across varying socioeconomic backgrounds among youths studied at multiple time points.

Thus, we aimed to better understand whether any pandemic-exacerbated mental health symptoms were more pronounced for those from lower socioeconomic status households using data from the Adolescent Brain Cognitive Development (ABCD) Study (data release 4.0), a 21-site, 10-year longitudinal cohort study of brain and cognitive development in approximately 12 000 demographically and geographically diverse youths. Enrollment for the baseline visit (ages 9 to 10 years) occurred between 2016 to 2018, whereas the 2-year follow-up (ages 11 to 12 years) halted data collection for some youths due to the COVID-19 lockdown. As some youths underwent the 2-year follow-up visit prior to the lockdown, this dataset provides the context of a natural experiment–like design to evaluate how development may have been associated with the pandemic. We hypothesized that the pandemic would be associated with worsening mental health, particularly for internalizing behaviors, such as anxiety and depression, and that these associations would be exacerbated in youths from lower socioeconomic status households.

## Methods

The ABCD Study design, recruitment, and protocols are published in numerous articles.^7^ A centralized institutional review board research approval was obtained from the University of California San Diego; written consent was obtained from caregivers, and verbal assent was provided by the youths. The current report follows the Strengthening the Reporting of Observational Studies in Epidemiology (STROBE) reporting guideline. We included measures obtained from the demographic questionnaires (including an income-to-needs ratio [INR]), Child Behavior Checklist (CBCL),^8^ and Family Environment Scale (FES) from the 1-year follow-up (aged 10 to 11 years) and 2-year follow-up (aged 11 to 12 years) appointments. This design allowed us to examine changes in mental health problems prior to and after the onset of the COVID-19 lockdown. Only youths who were missing data from the 2-year follow-up visits that were of interest to our study were excluded. This resulted in a final sample of 10 399 youths. Siblings were initially included in the analyses to be as inclusive as possible, and sensitivity tests were conducted to determine whether their inclusion was associated with different outcomes.

### Demographics

Caregivers reported on the youth’s sex at birth, date of birth (from which years in months was calculated), race and ethnicity, along with their education, annual household income, and number of people residing in the household. Twenty-nine options existed for caregivers to self-select their child’s race, which were collapsed into 6 categories (eg, American Indian or Alaska Native/Native Hawaiian or Pacific Islander, Asian, Black, multiracial, White, other [free answer question]) for descriptive purposes only. Two options were available for caregivers to self-select for their child’s ethnicity: (1) Latinx or Hispanic or (2) not Latinx or Hispanic. Caregiver highest education level (22 categories) was obtained for both the attending caregiver at the visit, as well as any other caregivers, and for analysis purposes, was collapsed into 5 categories (ie, below high school diploma, high school diploma/general education diploma, some college, bachelor’s degree, postgraduate degree). Total family income was collected using a 10-category scale representing various income ranges (eg, below $5000 to more than $200 000). An INR was calculated by dividing the total household income (ie, the mean income range of each category) at baseline (youths aged 9 to 10 years) by the poverty rate per household size, according to the 2017 federal poverty guidelines^9^; lower values indicate lower socioeconomic status. Baseline household income was used because this is fairly stable.

### Child Behavior Checklist

Caregivers completed the CBCL,^8^ which was designed to assess emotional and behavioral symptoms in youths aged 6 to 18 years. Item-level responses to this 113-item questionnaire consisted of 0 (not true), 1 (somewhat/sometimes true), or 2 (very true). Summary scores are available for 8 metrics: (1) anxious/depressed, (2) withdrawn/depressed, (3) somatic complaints, (4) social problems, (5) thought problems, (6) attention problems, (7) rule-breaking behavior, and (8) aggressive behavior. These scales form 2 overarching factors: (1) internalizing problems and (2) externalizing problems; while a total problems summary score is the summation across all 8 metrics. The internalizing factor consisted of a summed score of the anxious/depressed, withdrawn/depressed, and somatic complaints subscales. The externalizing score was a summed score of the rule-breaking and aggressive behavior subscales.

### Family Environment Scale

The FES^10^ is a validated assessment that was completed by the caregiver and youth. We focused on the 9-item conflict subscale, which assesses conflict among family members as reported by youth only. A summary score consists of the sum across each of the 9 questionnaire items.

### Classification of Prepandemic and Intrapandemic Groups

The prepandemic group was defined as having a 2-year follow-up visit before the COVID-19 lockdown (prior to March 11, 2020). The intrapandemic group was defined as having a 2-year follow-up visit after March 11, 2020 (ie, after lockdown restrictions were implemented, and data were either collected remotely or in person once lockdown restrictions were lifted). Although 10 399 youths had available data at the 2-year follow-up, 228 of these youths had missing data at the previous visit (ie, at the 1-year follow-up). This yielded a final sample of 10 171 youths with 1-year-follow-up data available (prepandemic n = 7343; intrapandemic n = 2828) and 10 399 youths with 2-year follow-up data available (prepandemic n = 7493; intrapandemic n = 2906); missing data are reported in eTable 1 in Supplement 1.

### Statistical Analysis

At the 1-year follow-up, demographic characteristics were compared between prepandemic and intrapandemic groups using χ^2^ tests for categorical variables and *t* tests for continuous variables. To examine the association between the COVID-19 pandemic lockdown and within-individual changes in CBCL outcomes from the 1-year (aged 10 to 11 years) to 2-year follow-up (aged 11 to 12 years), we conducted longitudinal linear mixed-effect models with a group (ie, prepandemic vs intrapandemic) × time (ie, 1-year vs 2-year) interaction term, and fixed effects for sex, age (months), INR, caregiver highest education level, interstudy interval (ie, time interval [months] between 1-year and 2-year follow-ups), as well as by participant and by study site random intercepts. Additionally, we assessed whether the association between the pandemic and the CBCL outcomes varied over time depending on INR by introducing a group × time × INR interaction term: CBCL ~ Group × Time + Group × Time × INR + Sex + Age (months) + Caregiver Highest Education + Interstudy Interval + (1|site) + (1 + paritcipant ID).

To control for familywise error rates when performing multiple comparison tests, we used the Benjamini-Hochberg method; only results that passed multiple comparisons tests were reported. Statistical significance was determined a priori at 2-sided *P* < .05. All analyses were conducted in Stata version 17 (StataCorp). Given previous studies have suggested that the COVID-19 lockdown may have exacerbated family conflict, our post hoc analyses aimed to explore whether our findings remained robust when considering youth reports of family conflict. Specifically, we examined whether the group × time × INR interaction effect changed when incorporating FES scores into the models.

## Results

Among the final sample of 10 399 youths, 3947 (52.3%) were male; 2084 (20.3%) were Latinx/Hispanic; 1491 (14.5%) were Black, 1252 (12.2%) were multiracial, and 6765 (66.0%) were White; 4600 (44.2%) reported caregiver education levels below a 4-year college degree; 2475 (26.2%) had INR either below 100% (indicating poverty) or between 100% and less than 200% (near poverty) (Table 1). At the 1-year follow-up, the 7493 individuals in the intrapandemic group were, on average, slightly younger than the 2906 individuals in the prepandemic group (mean [SD] age of intrapandemic group: 129.8 [7.7] months vs prepandemic group: 131.6 [7.7] months). Additionally, the intrapandemic group had a longer mean (SD) interstudy interval (16.1 [2.7] months) compared with the prepandemic group (11.7 [1.8] months). Consequently, at the 2-year follow-up, individuals in the intrapandemic group were, on average, slightly older than those in the prepandemic group (mean [SD] age of the intrapandemic group: 145.9 [8.1] months vs prepandemic group: 143.3 [7.8] months). Moreover, individuals in the intrapandemic group were more likely to be identified as Latinx/Hispanic (22.3% vs 19.5%) or Black (19.2% vs 12.7%), have caregivers with education below college level (46.7% vs 43.8%), come from families with lower income (16.5% vs 12.4%), and report a higher mean FES score (8.1% vs 7.8%) compared with individuals in the prepandemic group. At 1-year follow-up, youths in the intrapandemic group exhibited slightly higher average CBCL raw scores compared with those in the prepandemic group for anxious/depressed, somatic complaints, social problems, attention problems, rule-breaking behavior, aggressive behavior, internalizing, externalizing, and total problems. However, the averages for thought problems and withdrawn/depressed were more similar between the groups. Detailed information regarding the distributions of CBCL outcomes by groups can be found in Table 2.

**Table 1.** Participants Characteristics by Prepandemic and Intrapandemic Groups

Characteristics	Participants, No. (%)		
	Prepandemic (n = 7493 [72.1%])	Intrapandemic (n = 2906 [27.9%])	Total (N = 10 399)
Sex			
Female	3546 (47.3)	1411 (48.6)	4957 (47.7)
Male	3947 (52.7)	1495 (51.4)	5442 (52.3)
Ethnicity^a^			
Latinx/Hispanic	1443 (19.5)	641 (22.3)	2084 (20.3)
Non-Latinx/Hispanic	5954 (80.5)	2238 (77.7)	8192 (79.7)
Race^a^			
American Indian or Alaska Native/Native Hawaiian or Pacific Islander	49 (0.7)	17 (0.6)	66 (0.6)
Asian	180 (2.4)	56 (2.0)	236 (2.3)
Black	942 (12.7)	549 (19.2)	1491 (14.5)
Multiracial	843 (11.4)	409 (14.3)	1252 (12.2)
White	5084 (68.7)	1681 (58.9)	6765 (66.0)
Other^b^	303 (4.1)	141 (4.9)	444 (4.3)
Education^a^			
<HS diploma	412 (5.5)	190 (6.6)	602 (5.8)
HS diploma/GED	646 (8.7)	323 (11.2)	969 (9.4)
Some college	2199 (29.5)	830 (28.8)	3029 (29.3)
Bachelor’s degree	2227 (29.9)	796 (27.6)	3023 (29.2)
Postgraduate degree	1970 (26.4)	744 (25.8)	2714 (26.3)
Interstudy interval, mean (SD), mo^a^	11.7 (1.8)	16.1 (2.7)	12.9 (2.9)
INR categories^a^			
Poverty	849 (12.4)	429 (16.5)	1278 (13.5)
Near poverty	878 (12.8)	319 (12.3)	1197 (12.7)
Middle	3152 (46.1)	1148 (44.2)	4300 (45.6)
Upper	1966 (28.7)	699 (26.9)	2665 (28.2)
INR, mean (SD)^a,c^	3.9 (2.4)	3.7 (2.4)	3.9 (2.4)
Age, mean (SD), mo			
1-y follow-up (n = 10 171)	131.6 (7.7)	129.8 (7.7)	131.1 (7.7)
2-y follow-up (n = 10 399)	143.3 (7.8)	145.9 (8.1)	144.0 (8.0)
FES Scale raw score, mean (SD), points^a^			
1-y Follow-up (n = 10 171)	1.8 (1.8)	2.1 (2.0)	1.9 (1.9)
2-y Follow-up (n = 10 399)	1.9 (1.8)	2.0 (1.8)	1.9 (1.8)

Abbreviations: FES, Family Environment Scale; GED, Generalized Education Diploma; HS, high school; INR, income-to-needs ratio.

^a^ The frequency and percentage of incomplete variables: ethnicity (n = 123 [1.18%]), race (n = 145 [1.39%]); education (n = 89 [0.86%]); INR (n = 959 [9.22%]); FES raw score at 1-year follow-up (n = 4 [0.04%]); FES raw score at 2-year follow-up (n = 35 [0.34%]). Demographic characteristics were compared between prepandemic and intrapandemic groups using χ^2^ test for categorical variables and *t* test for continuous variables. Race and ethnicity were self-selected by the caregiver for the youth. There were 29 response options of which were combined into 6 grouping variables for descriptive purposes only.

^b^ The other race category was presented as free answer (no specific categories to choose from).

^c^ INR was used as a continuous variable in these analyses, but it is reported as a categorical and continuous for ease of interpretation.

**Table 2.** Distribution of Child Behavioral Checklist Outcomes Over Time by Prepandemic and Intrapandemic Groups^a^

Outcomes	Mean (SD)		
	Prepandemic (n = 7493 [72.1%])	Intrapandemic (n = 2906 [27.9%])	Total (N = 10 399)
**Total problems raw score**			
1-y Follow-up	17.2 (16.9)	18.1 (18.4)	17.4 (17.4)
2-y Follow-up	16.4 (17.0)	16.3 (17.4)	16.4 (17.0)
**Internalizing problems raw score**			
1-y Follow-up	5.0 (5.4)	5.3 (5.8)	5.1 (5.5)
2-y Follow-up	4.9 (5.6)	4.9 (5.6)	4.9 (5.6)
**Externalizing problems raw score**			
1-y Follow-up	4.0 (5.4)	4.4 (6.0)	4.1 (5.6)
2-y Follow-up	3.9 (5.5)	3.9 (5.9)	3.9 (5.5)
**Anxious/depressed raw score**			
1-y Follow-up	2.5 (3.0)	2.6 (3.3)	2.5 (3.1)
2-y Follow-up	2.3 (3.0)	2.2 (2.9)	2.3 (3.0)
**Aggressive behavior raw score**			
1-y Follow-up	3.0 (4.0)	3.2 (4.5)	3.0 (4.1)
2-y Follow-up	2.9 (4.0)	2.8 (4.3)	2.9 (4.0)
**Thought problems raw score**			
1-y Follow-up	1.6 (2.2)	1.6 (2.3)	1.6 (2.2)
2-y Follow-up	1.4 (2.1)	1.3 (2.0)	1.4 (2.1)
**Attention problems raw score**			
1-y Follow-up	2.8 (3.4)	2.9 (3.5)	2.8 (3.4)
2-y Follow-up	2.7 (3.3)	2.7 (3.4)	2.7 (3.3)
**Social problems raw score**			
1-y Follow-up	1.5 (2.1)	1.6 (2.2)	1.5 (2.2)
2-y Follow-up	1.3 (2.1)	1.4 (2.1)	1.3 (2.1)
**Rule-breaking behavior raw score**			
1-y Follow-up	1.1 (1.7)	1.2 (1.9)	1.1 (1.8)
2-y Follow-up	1.1 (1.8)	1.1 (1.9)	1.1 (1.8)
**Somatic complaints raw score**			
1-y Follow-up	1.4 (1.9)	1.5 (2.0)	1.5 (1.9)
2-y Follow-up	1.4 (1.9)	1.3 (1.9)	1.4 (1.9)
**Withdrawn/depressed raw score**			
1-y Follow-up	1.1 (1.7)	1.1 (1.8)	1.1 (1.8)
2-y Follow-up	1.2 (1.9)	1.3 (1.9)	1.2 (1.9)

^a^ The 1-year follow-up consisted of 10 171 individuals: 7343 in the prepandemic group and 2828 in the intrapandemic group; 2-year follow-up consisted of 10 399 individuals: 7493 in the prepandemic group and 2906 in the intrapandemic group. The frequency and percentage of incomplete variables: CBCL outcomes at 1-year follow-up were 14 (0.14%); CBCL outcomes at 2-year follow-up were 2329 (22.4%).

### INR Over Time Between CBCL Total Problems Among Youths in Prepandemic vs Intrapandemic Groups

While there was no significant evidence suggesting mean differences in CBCL Total Problems between youths in the prepandemic and intrapandemic groups over time, an increase of 1 unit in INR corresponded to a reduction in the average rate of change in Total Problems, with all other covariates held constant. Subsequent analysis including group × time × INR, found that INR moderated the group × time interactions (Table 3). This implies that, on average, greater total problems over time was associated with higher INR when comparing youths in the intrapandemic group with the prepandemic group. Specifically, when comparing individuals who differed by 1 unit on INR between prepandemic and intrapandemic groups from 1-year to 2-year follow-up, their expected difference in total problems score was 0.79 (95% CI, 0.37-1.22; false discovery rate [FDR]-corrected *P* < .001), holding sex, age, caregiver education, and interstudy interval constant (Figure). This finding suggests that total problems tend to be lower among youths from families with lower INR who experience the pandemic compared with those in the prepandemic group who had their follow-up visit prior to the pandemic. Further post hoc analysis suggests that after accounting for youth reports of family conflict (FES) and removal of siblings, the original findings remained unchanged.

**Table 3.** Estimated Regression Coefficient From Mixed-Effects Regression Analysis of Child Behavioral Checklist Total Problems Raw Score Between Prepandemic and Intrapandemic Groups Modified by INR, Adjusting for Interstudy Interval, Education, Age, and Sex^a^

Variables	Coefficient (SE) [95% CI]	*P* value
Age	−0.01 (0.02) [−0.05 to 0.04]	.79
Sex		
Female	0	<.001
Male	2.69 (0.33) [2.04 to 3.34]	
Education		
<High school diploma	0	NA
High school diploma/GED	−0.54 (1.00) [−2.50 to 1.41]	.59
Some college	1.79 (0.88) [0.07 to 3.51]	.04
Bachelor’s degree	0.05 (0.92) [−1.75 to 1.85]	.96
Postgraduate degree	−1.01 (0.95) [−2.87 to 0.85]	.29
Interstudy interval (months)	0.19 (0.09) [0.02 to 0.36]	.03
INR^b^	−0.70 (0.10) [−0.90 to −0.50]	<.001
Group		
Prepandemic	0	.37
Intrapandemic	−0.73 (0.82) [−2.33 to 0.87]	
Time		
Year 1	0	.004
Year 2	−1.05 (0.37) [−1.76 to −0.33]	
Group × time		
Prepandemic × year 1	0	.002
Prepandemic × year 2	0	
Intrapandemic × year 1	0	
Intrapandemic × year 2	−3.04 (0.99) [−4.98 to −1.11]	
Group × INR		
Prepandemic	0	.65
Intrapandemic	0.07 (0.16) [−0.25 to 0.39]	
Time × INR		
Year 1	0	.18
Year 2	0.08 (0.06) [−0.04 to 0.20]	
Group × time × INR		
Prepandemic × year 1	0	<.001
Prepandemic × year 2	0	
Intrapandemic × year 1	0	
Intrapandemic × year 2	0.79 (0.22) [0.37 to 1.22]	

Abbreviations: GED, Generalized Education Diploma; INR, income-to-needs ratio.

^a^ Demographic characteristics were compared between prepandemic and intrapandemic groups using χ^2^ test for categorical variables and *t* test for continuous variables. Year 1 was collected at ages 10 or 11 years; year 2 was collected at ages 11 or 12 years.

^b^ INR was used as a continuous variable in our analyses, but we report it as a categorical and continuous for ease of interpretation.

**Figure.**
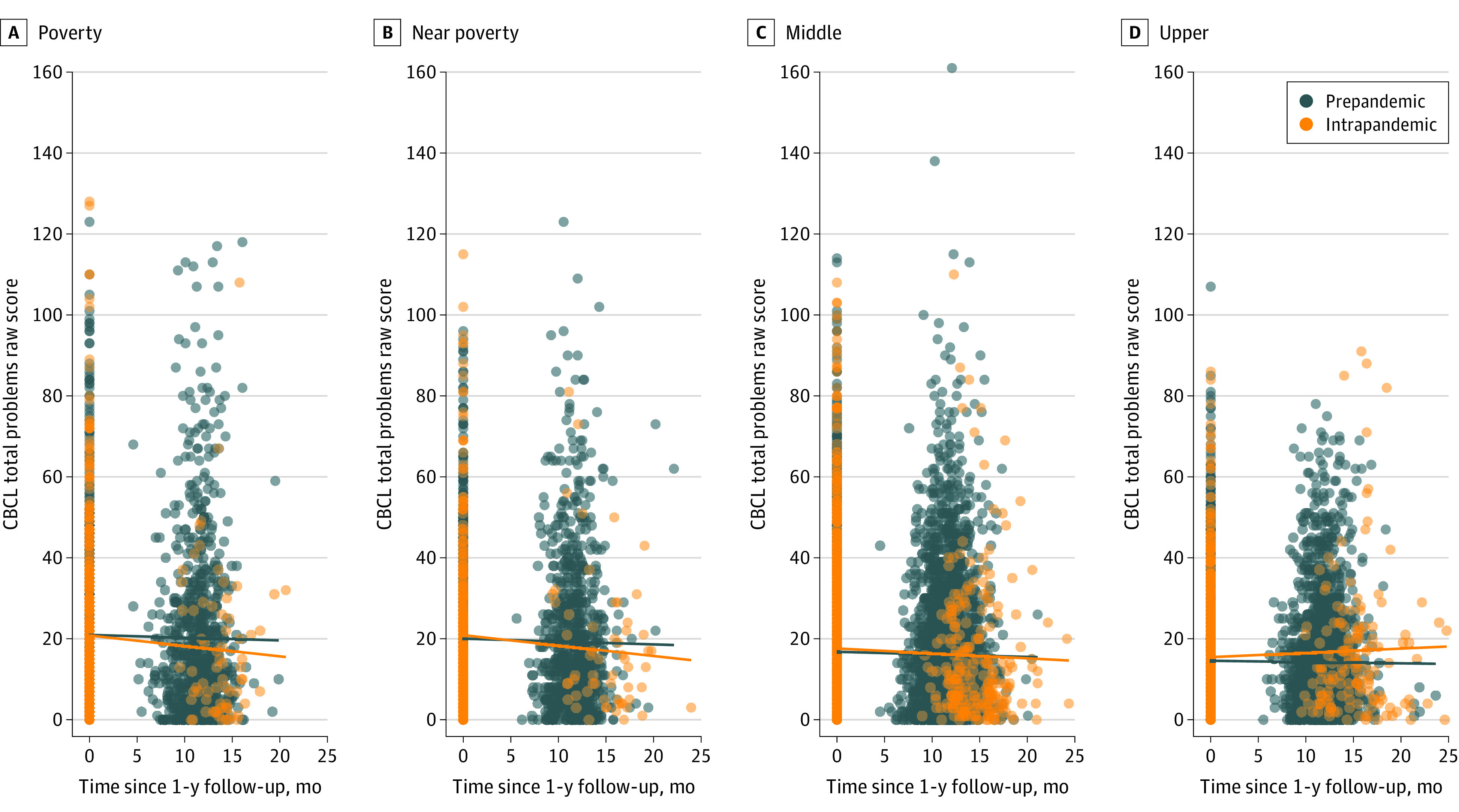
Association of INR and Total Problems Raw Score by Group Each panel shows associations between CBCL total problems at 1-year and 2-year follow-ups by different income-to-needs ratio (INR) category (poverty: <100%; near poverty: 100% to < 200%; middle: 200% to <600%; upper: ≥600%). The observed data points (circles) and best-fitted regression lines are shown for prepandemic and intrapandemic groups. The figure indicates reduced behavioral issues among individuals exposed to pandemic in the poverty category, and conversely, elevated problems in upper-income category. The figure prominently demonstrates a reduction over time in total problems among youths from families experiencing poverty and pandemic exposure compared with those in the prepandemic group.

### INR Over Time Between Specific CBCL Behavioral Problems Among Youths in Prepandemic vs Intrapandemic Groups

Similarly, no significant differences in mean CBCL behavioral problems for each subscale were observed between groups over time (group × time); however, while holding other variables constant, higher INR was associated with improved behavior problem scores (eg, anxious/depressed, aggressive behavior, attention problems, social problems, rule-breaking behavior, thought problems, internalizing and externalizing problems). In subsequent analyses, INR moderated the association between group × time for most of the CBCL subscales (eTables 2-9, eFigures 1-8 in Supplement 1), except for Withdrawn/Depressed and Somatic Complaints (eTables 10 and 11, eFigure 9 and 10 in Supplement 1). In particular, for every 1-unit difference in INR, the expected differences between intrapandemic and prepandemic groups from 1-year to 2-year follow-up were 0.19 (95% CI, 0.10-0.27; FDR-corrected *P* = .005) for anxious/depressed; 0.17 (95% CI, 0.06-0.28; FDR-corrected *P* = .03) for aggressive behavior; 0.09 (95% CI, 0.00-0.17; FDR-corrected *P* = .04) for attention problems; 0.08 (95% CI, 0.02-0.14; FDR-corrected *P* = .03) for social problems; 0.06 (95% CI, 0.01-0.11; FDR-corrected *P* = .04) for rule-breaking behavior; 0.12 (95% CI, 0.06-0.18; FDR-corrected *P* = .01) for thought problems; 0.27 (95% CI, 0.11-0.42; FDR-corrected *P* = .02) for internalizing problems; and 0.23 (95% CI, 0.09-0.37; FDR-corrected *P* = .02) for externalizing problems. These results suggest that most behavioral outcomes appear to be less negative in change over time among youths from families with lower INR who were also exposed to the pandemic, compared with those in the prepandemic group (see eFigures 1-10 in Supplement 1). Based on the post hoc analysis, controlling for FES did not change the observed associations. Sensitivity analyses indicated that removing siblings from the analyses did not affect the results.

## Discussion

Generally, families with lower income were more likely to experience health consequences due to COVID-19,^11^ which suggests that they may also be disproportionately indirectly affected by other psychosocial stressors (eg, increased mental health problems) that stemmed from the COVID-19 lockdown. Our study used a natural experimental design to examine whether mental health problems worsened for youths aged 10 to 12 years due to the COVID-19 lockdown and whether this was exacerbated by socioeconomic status (ie, INR). Contrary to our hypothesis, among youths from higher socioeconomic status households, the COVID-19 lockdown was associated with worsening emotional and behavioral symptoms, whereas youths in families with lower socioeconomic status had better outcomes. Importantly, these associations were not driven by the well-documented increases in family conflict during lockdown.^3,12^ In other words, our data suggest that youths from higher- vs lower-income families may have been disproportionately negatively affected by the COVID-19 lockdown in terms of their emotional and behavioral health.

Some data suggest that the COVID-19 lockdown had disproportionately negative correlations in families with higher income. For example, from April to June 2019 to April 2020, individuals of higher socioeconomic status reported a greater decline in life satisfaction than those with lower socioeconomic statuses.^11^ Moreover, Scrimin et al^13^ found that relationships between parental distress and child discomfort were only present in households with higher socioeconomic statuses, such that children in families with lower incomes may have more readily adapted to the added stressors of the pandemic. Indeed, our research group previously published data demonstrating that families with more socioeconomic disadvantage may have taken more action to alleviate emotional distress related to the COVID-19 pandemic.^14^ Thus, congruent with the shift-and-persist model, which describes increased resilience in more distressed families,^15^ our results suggest that youths in lower-income families may have demonstrated greater emotional and behavioral resilience or been less detrimentally affected by the COVID-19 lockdown.

### Limitations

The strength of this study lies in its natural experiment design, where we were able to examine within-person associations between mental health changes before and after the COVID-19 lockdown. However, this study has limitations. The second phase of recruitment for the ABCD Study contained more youths from lower socioeconomic backgrounds and groups who have historically not been included and/or participated in research. As such, the demographics of the intrapandemic group reflect this. Relatedly, the COVID-19 pandemic and lockdown restrictions differentially affected groups from various socioeconomic and specific cultural groups, which could lead to bias in representation of our results. Notably, this study cannot provide insight into causal pathways.

## Conclusions

Although causal inference is not possible, this study capitalizes on the strengths of a natural experimental design to examine COVID-19–associated changes in emotional and behavioral symptoms in youths. We highlight potential disparities in emotional and behavioral health among youths from various socioeconomic status backgrounds related to the pandemic, thus providing strong rationale for further investigation. However, this study cannot provide insight into the mechanistic drivers of differences between intrapandemic and prepandemic groups due to the lack of comprehensive data to measure these associations (eg, assessments of school vs home environment). Therefore, we are limited in our ability to offer interpretation as to why emotional and behavioral symptoms were differentially associated with time and exposure to the pandemic among youths from lower socioeconomic backgrounds. Ongoing assessments in ABCD will allow researchers to determine if decrements in emotional and behavioral health persist in the post–COVID-19 era.

## Notes

Supplement 1. **eTable 1.** Frequency and Percentage of Missing Data at 1-Year and 2-Year Follow-Ups **eTable 2.** Results From the Regression Analysis of the CBCL Anxious/Depressed Subscale **eTable 3.** Results From the Regression Analysis of the CBCL Aggressive Behavior Subscale **eTable 4.** Results From the Regression Analysis of the CBCL Attention Problems Subscale **eTable 5.** Results From the Regression Analysis of the CBCL Social Problems Subscale **eTable 6.** Results From the Regression Analysis of the CBCL Rule-Breaking Behavior Subscale **eTable 7.** Results From the Regression Analysis of the CBCL Thought Problems Subscale **eTable 8.** Results From the Regression Analysis of the CBCL Internalizing Problems Subscale **eTable 9.** Results From the Regression Analysis of the CBCL Externalizing Problems Subscale **eTable 10.** Results From the Regression Analysis of the CBCL Withdrawn/Depressed Subscale **eTable 11.** Results From the Regression Analysis of the CBCL Somatic Complaints Subscale **eFigure 1.** Associations of the Anxious/Depressed Subscale and INR Over Time by Group **eFigure 2.** Associations of the Aggressive Behavior Subscale and INR Over Time by Group **eFigure 3.** Associations of the Attention Problems Subscale and INR Over Time by Group **eFigure 4.** Associations of the Social Problems Subscale and INR Over Time by Group **eFigure 5.** Associations of the Rule-Breaking Subscale and INR Over Time by Group **eFigure 6.** Associations of the Thought Problems Subscale and INR Over Time by Group **eFigure 7.** Associations of the Internalizing Problems Subscale and INR Over Time by Group **eFigure 8.** Associations of the Externalizing Problems Subscale and INR Over Time by Group **eFigure 9.** Associations of the Withdrawn/Depressed Subscale and INR Over Time by Group **eFigure 10.** Associations of the Somatic Complaints Subscale and INR Over Time by Group

Supplement 2.
Data Sharing Statement
