# Supplementary material for: Socioeconomic Disadvantage and Youth Mental Health During the COVID-19 Pandemic Lockdown
Source: JAMA Netw Open. 2024 Jul 5;7(7):e2420466. doi: 10.1001/jamanetworkopen.2024.20466 (PMC11227076; doi:10.1001/jamanetworkopen.2024.20466)
Supplement: Supplement 1. — eTable 1. Frequency and Percentage of Missing Data at 1-Year and 2-Year Follow-Ups eTable 2. Results From the Regression Analysis of the CBCL Anxious/Depressed Subscale eTable 3. Results From the Regression Analysis of the CBCL Aggressive Behavior Subscale eTable 4. Results From the Regression Analysis of the CBCL Attention Problems Subscale eTable 5. Results From the Regression Analysis of the CBCL Social Problems Subscale eTable 6. Results From the Regression Analysis of the CBCL Rule-Breaking Behavior Subscale eTable 7. Results From the Regression Analysis of the CBCL Thought Problems Subscale eTable 8. Results From the Regression Analysis of the CBCL Internalizing Problems Subscale eTable 9. Results From the Regression Analysis of the CBCL Externalizing Problems Subscale eTable 10. Results From the Regression Analysis of the CBCL Withdrawn/Depressed Subscale eTable 11. Results From the Regression Analysis of the CBCL Somatic Complaints Subscale eFigure 1. Associations of the Anxious/Depressed Subscale and INR Over Time by Group eFigure 2. Associations of the Aggressive Behavior Subscale and INR Over Time by Group eFigure 3. Associations of the Attention Problems Subscale and INR Over Time by Group eFigure 4. Associations of the Social Problems Subscale and INR Over Time by Group eFigure 5. Associations of the Rule-Breaking Subscale and INR Over Time by Group eFigure 6. Associations of the Thought Problems Subscale and INR Over Time by Group eFigure 7. Associations of the Internalizing Problems Subscale and INR Over Time by Group eFigure 8. Associations of the Externalizing Problems Subscale and INR Over Time by Group eFigure 9. Associations of the Withdrawn/Depressed Subscale and INR Over Time by Group eFigure 10. Associations of the Somatic Complaints Subscale and INR Over Time by Group [file jamanetwopen-e2420466-s001.pdf]

Supplemental Online Content

Adise S, West AE, Rezvan PH, et al. Socioeconomic disadvantage and youth mental health during the COVID-19 pandemic lockdown. *JAMA Netw Open.* 2024;7(7):e2420466. doi:10.1001/jamanetworkopen.2024.20466

eTable 1. Frequency and Percentage of Missing Data at 1-Year and 2-Year Follow-Ups

eTable 2. Results From the Regression Analysis of the CBCL Anxious/Depressed Subscale

eTable 3. Results From the Regression Analysis of the CBCL Aggressive Behavior Subscale

eTable 4. Results From the Regression Analysis of the CBCL Attention Problems Subscale

eTable 5. Results From the Regression Analysis of the CBCL Social Problems Subscale

eTable 6. Results From the Regression Analysis of the CBCL Rule-Breaking Behavior Subscale

eTable 7. Results From the Regression Analysis of the CBCL Thought Problems Subscale

eTable 8. Results from the regression analysis of the CBCL Internalizing Problems subscale

eTable 9. Results From the Regression Analysis of the CBCL Externalizing Problems Subscale

eTable 10. Results From the Regression Analysis of the CBCL Withdrawn/Depressed Subscale

eTable 11. Results From the Regression Analysis of the CBCL Somatic Complaints Subscale

eFigure 1. Associations of the Anxious/Depressed Subscale and INR Over Time by Group

eFigure 2. Associations of the Aggressive Behavior subscale and INR over time by Group

eFigure 3. Associations of the Attention Problems Subscale and INR Over Time by Group

© 2024 Adise S et al. *JAMA Network Open.*

eFigure 4. Associations of the Social Problems Subscale and INR Over Time by Group

eFigure 5. Associations of the Rule-Breaking Subscale and INR Over Time by Group

eFigure 6. Associations of the Thought Problems Subscale and INR Over Time by Group

eFigure 7. Associations of the Internalizing Problems Subscale and INR Over Time by Group

eFigure 8. Associations of the Externalizing Problems Subscale and INR Over Time by Group

eFigure 9. Associations of the Withdrawn/Depressed Subscale and INR Over Time by Group

eFigure 10. Associations of the Somatic Complaints Subscale and INR Over Time by Group

This supplemental material has been provided by the authors to give readers additional information about their work.

© 2024 Adise S et al. *JAMA Network Open.*

eTable1: Frequency and percentage of missing data at 1-year and 2-year follow-ups.

	1-year follow-up (n = 10,171)		2-year follow-up (n = 10,399)	
Variable	n	%	n	%
Group	0	0	0	0
Sex	0	0	0	0
Ethnicity	119	(1.17)	123	(1.18)
Race	136	(1.34)	145	(1.39)
Education[§]	35	0.34	1,661	15.97
Inter-study interval (months)	0	0	228	2.19
INR	913	8.98	959	9.22
Age (months)	0	0	0	0
FES Scale - Raw score	4	0.04	35	0.34
Anxious/Depressed raw score	14	0.14	2,329	22.4
Withdrawn/Depressed raw score	14	0.14	2,329	22.4
Somatic Complaints raw score	14	0.14	2,329	22.4
Social Problems raw score	14	0.14	2,329	22.4
Thought Problems raw score	14	0.14	2,329	22.4
Attention Problems raw score	14	0.14	2,329	22.4
Rule-Breaking Behavior raw score	14	0.14	2,329	22.4
Aggressive Behavior raw score	14	0.14	2,329	22.4
Internalizing Problems raw score	14	0.14	2,329	22.4
Externalizing Problems raw score	14	0.14	2,329	22.4
Total Problems raw score	14	0.14	2,329	22.4

Note. INR = income-to-needs ratio; FES = family environment scale. [§]The Missing Education data is derived from the original data. Missing values at the 2-year follow- up were replaced by carrying forward the 1-year follow-up data, leading to 32 (0.31) and 57 (0.55) missing education values at 1- and 2-year follow-ups, respectively. INR = income-to-needs ratio; FES = family environment scale.

© 2024 Adise S et al. *JAMA Network Open.*

eTable2: The estimated regression coefficient with 95% CI from mixed-effects regression analysis of CBCL Anxious/Depressed raw score between pre- and intra-pandemic groups modified by INR, adjusting for inter-study interval, education, age, and sex.

Variable	Coef.	SE	[95% CI]	p-value
Age	0.00	0.00	[-0.01, 0.01]	0.5468
Sex				
Female	0.00	0.00		
Male	-0.19	0.06	[-0.31, -0.08]	0.0010
Education				
< High school (HS) Diploma	0.00	0.00		
HS Diploma/GED	-0.21	0.18	[-0.56, 0.13]	0.2265
Some College	0.32	0.16	[0.02, 0.62]	0.0393
Bachelor's Degree	0.32	0.16	[0.00, 0.64]	0.0476
Postgraduate Degree	0.33	0.17	[0.00, 0.66]	0.0467
Inter-study interval (months)	0.03	0.02	[0.00, 0.06]	0.0302
INR	-0.06	0.02	[-0.09, -0.02]	0.0012
Group				
Pre-pandemic	0.00	0.00		
Intra-pandemic	-0.24	0.15	[-0.52, 0.05]	0.1051
Time				
Year 1	0.00	0.00		
Year 2	-0.25	0.07	[-0.39, -0.11]	0.0005
Group x Time				
Pre-pandemic x Year 1	0.00	0.00		
Pre-pandemic x Year 2	0.00	0.00		
Intra-pandemic x Year 1	0.00	0.00		
Intra-pandemic x Year 2	-0.81	0.20	[-1.21, -0.41]	0.0001
Group x INR				
Pre-pandemic	0.00	0.00		
Intra-pandemic	0.05	0.03	[-0.01, 0.11]	0.0910
Time x INR				
Year 1	0.00	0.00		
Year 2	0.01	0.01	[-0.01, 0.04]	0.3973
Group x Time x INR				
Pre-pandemic x Year 1	0.00	0.00		
Pre-pandemic x Year 2	0.00	0.00		
Intra-pandemic x Year 1	0.00	0.00		
Intra-pandemic x Year 2	0.19	0.04	[0.10, 0.27]	0.0000

Note. GED = Generalized Education Diploma; INR = income-to-needs ratio

© 2024 Adise S et al. *JAMA Network Open.*

eTable3: The estimated regression coefficient with 95% CI from mixed-effects regression analysis of CBCL Aggressive Behavior raw score between pre- and intra-pandemic groups modified by INR, adjusting for inter-study interval, education, age, and sex.

Variable	Coef.	SE	[95% CI]	p-value
Age	-0.00	0.00	[-0.01, 0.01]	0.4419
Sex				
Female	0.00	0.00		
Male	0.64	0.08	[0.49, 0.80]	0.0000
Education				
< High school (HS) Diploma	0.00	0.00		
HS Diploma/GED	-0.03	0.24	[-0.50, 0.43]	0.8827
Some College	0.24	0.21	[-0.17, 0.64]	0.2561
Bachelor's Degree	-0.05	0.22	[-0.47, 0.38]	0.8232
Postgraduate Degree	-0.20	0.22	[-0.64, 0.24]	0.3819
Inter-study interval (months)	0.04	0.02	[-0.00, 0.08]	0.0791
INR	-0.20	0.02	[-0.25, -0.15]	0.0000
Group				
Pre-pandemic	0.00	0.00		
Intra-pandemic	-0.06	0.19	[-0.44, 0.32]	0.7612
Time				
Year 1	0.00	0.00		
Year 2	-0.13	0.09	[-0.31, 0.05]	0.1440
Group x Time				
Pre-pandemic x Year 1	0.00	0.00		
Pre-pandemic x Year 2	0.00	0.00		
Intra-pandemic x Year 1	0.00	0.00		
Intra-pandemic x Year 2	-0.83	0.25	[-1.32, -0.35]	0.0008
Group x INR				
Pre-pandemic	0.00	0.00		
Intra-pandemic	0.02	0.04	[-0.05, 0.10]	0.5494
Time x INR				
Year 1	0.00	0.00		
Year 2	0.02	0.02	[-0.01, 0.05]	0.1306
Group x Time x INR				
Pre-pandemic x Year 1	0.00	0.00		
Pre-pandemic x Year 2	0.00	0.00		
Intra-pandemic x Year 1	0.00	0.00		
Intra-pandemic x Year 2	0.17	0.05	[0.06, 0.28]	0.0019

Note. GED = Generalized Education Diploma; INR = income-to-needs ratio

© 2024 Adise S et al. *JAMA Network Open.*

eTable4: The estimated regression coefficient with 95% CI from mixed-effects regression analysis of CBCL Attention Problems raw score between pre- and intra-pandemic groups modified by INR, adjusting for inter-study interval, education, age, and sex.

Variable	Coef.	SE	[95% CI]	p-value
Age	-0.01	0.00	[-0.01, 0.00]	0.2218
Sex				
Female	0.00	0.00		
Male	1.04	0.06	[0.91, 1.17]	0.0000
Education				
< High school (HS) Diploma	0.00	0.00		
HS Diploma/GED	-0.02	0.20	[-0.40, 0.36]	0.9203
Some College	0.39	0.17	[0.05, 0.73]	0.0234
Bachelor's Degree	0.12	0.18	[-0.24, 0.47]	0.5201
Postgraduate Degree	-0.08	0.19	[-0.44, 0.29]	0.6857
Inter-study interval (months)	0.02	0.02	[-0.01, 0.06]	0.1474
INR	-0.08	0.02	[-0.12, -0.04]	0.0000
Group				
Pre-pandemic	0.00	0.00		
Intra-pandemic	-0.08	0.16	[-0.40, 0.23]	0.5961
Time				
Year 1	0.00	0.00		
Year 2	-0.12	0.07	[-0.27, 0.02]	0.0847
Group x Time				
Pre-pandemic x Year 1	0.00	0.00		
Pre-pandemic x Year 2	0.00	0.00		
Intra-pandemic x Year 1	0.00	0.00		
Intra-pandemic x Year 2	-0.17	0.19	[-0.55, 0.21]	0.3878
Group x INR				
Pre-pandemic	0.00	0.00		
Intra-pandemic	-0.02	0.03	[-0.08, 0.05]	0.6295
Time x INR				
Year 1	0.00	0.00		
Year 2	0.01	0.01	[-0.01, 0.03]	0.3420
Group x Time x INR				
Pre-pandemic x Year 1	0.00	0.00		
Pre-pandemic x Year 2	0.00	0.00		
Intra-pandemic x Year 1	0.00	0.00		
Intra-pandemic x Year 2	0.09	0.04	[0.00, 0.17]	0.0379

Note. GED = Generalized Education Diploma; INR = income-to-needs ratio.

© 2024 Adise S et al. *JAMA Network Open.*

eTable5: The estimated regression coefficient with 95% CI from mixed-effects regression analysis of CBCL Social Problems raw score between pre- and intra-pandemic groups modified by INR, adjusting for inter-study interval, education, age, and sex.

Variable	Coef.	SE	[95% CI]	p-value
Age	-0.01	0.00	[-0.01, -0.00]	0.0091
Sex				
Female	0.00	0.00		
Male	0.12	0.04	[0.04, 0.20]	0.0034
Education				
< High school (HS) Diploma	0.00	0.00		
HS Diploma/GED	-0.23	0.12	[-0.47, 0.01]	0.0586
Some College	-0.06	0.11	[-0.27, 0.15]	0.5917
Bachelor's Degree	-0.30	0.11	[-0.52, -0.08]	0.0081
Postgraduate Degree	-0.41	0.12	[-0.63, -0.18]	0.0005
Inter-study interval	0.19	0.13	[-0.05, 0.44]	0.1206
INR	-0.09	0.01	[-0.11, -0.06]	0.0000
Group				
Pre-pandemic	0.00	0.00		
Intra-pandemic	-0.02	0.10	[-0.21, 0.18]	0.8721
Time				
Year 1	0.00	0.00		
Year 2	-0.11	0.05	[-0.20, -0.01]	0.0329
Group x Time				
Pre-pandemic x Year 1	0.00	0.00		
Pre-pandemic x Year 2	0.00	0.00		
Intra-pandemic x Year 1	0.00	0.00		
Intra-pandemic x Year 2	-0.29	0.14	[-0.56, -0.01]	0.0415
Group x INR				
Pre-pandemic	0.00	0.00		
Intra-pandemic	0.00	0.02	[-0.04, 0.04]	0.8352
Time x INR				
Year 1	0.00	0.00		
Year 2	0.01	0.01	[-0.00, 0.03]	0.1481
Group x Time x INR				
Pre-pandemic x Year 1	0.00	0.00		
Pre-pandemic x Year 2	0.00	0.00		
Intra-pandemic x Year 1	0.00	0.00		
Intra-pandemic x Year 2	0.08	0.03	[0.02, 0.14]	0.0095

Note. GED = Generalized Education Diploma; INR = income-to-needs ratio.

© 2024 Adise S et al. *JAMA Network Open.*

eTable6: The estimated regression coefficient with 95% CI from mixed-effects regression analysis of CBCL Rule-Breaking Behavior raw score between pre- and intra-pandemic groups modified by INR, adjusting for inter-study interval, education, age, and sex.

Variable	Coef.	SE	[95% CI]	p-value
Age	0.00	0.00	[-0.00, 0.01]	0.1499
Sex				
Female	0.00	0.00		
Male	0.38	0.03	[0.32, 0.45]	0.0000
Education				
< High school (HS) Diploma	0.00	0.00		
HS Diploma/GED	0.01	0.10	[-0.19, 0.20]	0.9592
Some College	0.07	0.09	[-0.10, 0.24]	0.4090
Bachelor's Degree	-0.19	0.09	[-0.37, -0.01]	0.0371
Postgraduate Degree	-0.31	0.09	[-0.49, -0.12]	0.0011
Inter-study interval (months)	0.01	0.01	[-0.01, 0.03]	0.1841
INR	-0.08	0.01	[-0.10, -0.06]	0.0000
Group				
Pre-pandemic	0.00	0.00		
Intra-pandemic	0.04	0.08	[-0.12, 0.20]	0.6388
Time				
Year 1	0.00	0.00		
Year 2	-0.09	0.04	[-0.17, -0.01]	0.0297
Group x Time				
Pre-pandemic x Year 1	0.00	0.00		
Pre-pandemic x Year 2	0.00	0.00		
Intra-pandemic x Year 1	0.00	0.00		
Intra-pandemic x Year 2	-0.19	0.12	[-0.42, 0.05]	0.1159
Group x INR				
Pre-pandemic	0.00	0.00		
Intra-pandemic	-0.01	0.02	[-0.04, 0.02]	0.5332
Time x INR				
Year 1	0.00	0.00		
Year 2	0.01	0.01	[-0.01, 0.02]	0.2210
Group x Time x INR				
Pre-pandemic x Year 1	0.00	0.00		
Pre-pandemic x Year 2	0.00	0.00		
Intra-pandemic x Year 1	0.00	0.00		
Intra-pandemic x Year 2	0.06	0.03	[0.01, 0.11]	0.0225

Note. GED = Generalized Education Diploma; INR = income-to-needs ratio.

© 2024 Adise S et al. *JAMA Network Open.*

eTable7: Table estimated regression coefficient with 95% CI from mixed-effects regression analysis of CBCL Thought Problems raw score between pre- and intra-pandemic groups modified by INR, adjusting for inter-study interval, education, age, and sex.

Variable	Coef.	SE	[95% CI]	p-value
Age	-0.00	0.00	[-0.01, 0.00]	0.3389
Sex				
Female	0.00	0.00		
Male	0.31	0.04	[0.23, 0.39]	0.0000
Education				
< High school (HS) Diploma	0.00	0.00		
HS Diploma/GED	0.11	0.12	[-0.14, 0.35]	0.3923
Some College	0.28	0.11	[0.07, 0.49]	0.0096
Bachelor's Degree	0.16	0.11	[-0.06, 0.38]	0.1625
Postgraduate Degree	0.00	0.12	[-0.23, 0.23]	0.9882
Inter-study interval (months)	0.02	0.01	[-0.00, 0.04]	0.0959
INR	-0.02	0.01	[-0.05, 0.00]	0.0747
Group				
Pre-pandemic	0.00	0.00		
Intra-pandemic	-0.03	0.10	[-0.23, 0.17]	0.7521
Time				
Year 1	0.00	0.00		
Year 2	-0.11	0.05	[-0.21, -0.01]	0.0368
Group x Time				
Pre-pandemic x Year 1	0.00	0.00		
Pre-pandemic x Year 2	0.00	0.00		
Intra-pandemic x Year 1	0.00	0.00		
Intra-pandemic x Year 2	-0.52	0.14	[-0.81, -0.24]	0.0003
Group x INR				
Pre-pandemic	0.00	0.00		
Intra-pandemic	-0.01	0.02	[-0.05, 0.03]	0.5904
Time x INR				
Year 1	0.00	0.00		
Year 2	-0.01	0.01	[-0.02, 0.01]	0.5443
Group x Time x INR				
Pre-pandemic x Year 1	0.00	0.00		
Pre-pandemic x Year 2	0.00	0.00		
Intra-pandemic x Year 1	0.00	0.00		
Intra-pandemic x Year 2	0.12	0.03	[0.06, 0.18]	0.0002

Note. GED = Generalized Education Diploma; INR = income-to-needs ratio.

© 2024 Adise S et al. *JAMA Network Open.*

eTable8: The estimated regression coefficient with 95% CI from mixed-effects regression analysis of CBCL Internalizing Problems raw score between pre- and intra-pandemic groups modified by INR, adjusting for inter-study interval, education, age, and sex.

Variable	Coef.	SE	[95% CI]	p-value
Age	0.02	0.01	[0.00, 0.03]	0.0107
Sex				
Female	0.00	0.00		
Male	-0.36	0.11	[-0.57, -0.15]	0.0007
Education				
< High school (HS) Diploma	0.00	0.00		
HS Diploma/GED	-0.39	0.32	[-1.02, 0.24]	0.2208
Some College	0.51	0.28	[-0.04, 1.07]	0.0689
Bachelor's Degree	0.27	0.30	[-0.31, 0.85]	0.3682
Postgraduate Degree	0.14	0.31	[-0.46, 0.74]	0.6551
Inter-study interval (months)	0.06	0.03	[0.01, 0.12]	0.0247
INR	-0.16	0.03	[-0.23, -0.10]	0.0000
Group				
Pre-pandemic	0.00	0.00		
Intra-pandemic	-0.41	0.27	[-0.93, 0.11]	0.1252
Time				
Year 1	0.00	0.00		
Year 2	-0.40	0.13	[-0.66, -0.15]	0.0019
Group x Time				
Pre-pandemic x Year 1	0.00	0.00		
Pre-pandemic x Year 2	0.00	0.00		
Intra-pandemic x Year 1	0.00	0.00		
Intra-pandemic x Year 2	-1.11	0.36	[-1.83, -0.40]	0.0023
Group x INR				
Pre-pandemic	0.00	0.00		
Intra-pandemic	0.07	0.05	[-0.04, 0.17]	0.2061
Time x INR				
Year 1	0.00	0.00		
Year 2	0.02	0.02	[-0.02, 0.06]	0.3502
Group x Time x INR				
Pre-pandemic x Year 1	0.00	0.00		
Pre-pandemic x Year 2	0.00	0.00		
Intra-pandemic x Year 1	0.00	0.00		
Intra-pandemic x Year 2	0.27	0.08	[0.11, 0.42]	0.0009

Note. GED = Generalized Education Diploma; INR = income-to-needs ratio.

© 2024 Adise S et al. *JAMA Network Open.*

eTable9: The estimated regression coefficient with 95% CI from mixed-effects regression analysis of CBCL Externalizing Problems raw score between pre- and intra-pandemic groups modified by INR, adjusting for inter-study interval, education, age, and sex.

Variable	Coef.	SE	[95% CI]	p-value
Age	-0.00	0.01	[-0.01, 0.01]	0.9294
Sex				
Female	0.00	0.00		
Male	1.03	0.11	[0.82, 1.23]	0.0000
Education				
< High school (HS) Diploma	0.00	0.00		
HS Diploma/GED	-0.03	0.32	[-0.66, 0.59]	0.9129
Some College	0.30	0.28	[-0.24, 0.85]	0.2775
Bachelor's Degree	-0.24	0.29	[-0.82, 0.33]	0.4023
Postgraduate Degree	-0.51	0.30	[-1.10, 0.08]	0.0913
Inter-study interval (months)	0.05	0.03	[-0.01, 0.10]	0.0847
INR	-0.28	0.03	[-0.34, -0.22]	0.0000
Group				
Pre-pandemic	0.00	0.00		
Intra-pandemic	-0.02	0.26	[-0.53, 0.49]	0.9378
Time				
Year 1	0.00	0.00		
Year 2	-0.23	0.12	[-0.46, 0.01]	0.0612
Group x Time				
Pre-pandemic x Year 1	0.00	0.00		
Pre-pandemic x Year 2	0.00	0.00		
Intra-pandemic x Year 1	0.00	0.00		
Intra-pandemic x Year 2	-1.01	0.33	[-1.66, -0.37]	0.0021
Group x INR				
Pre-pandemic	0.00	0.00		
Intra-pandemic	0.01	0.05	[-0.09, 0.12]	0.8050
Time x INR				
Year 1	0.00	0.00		
Year 2	0.03	0.02	[-0.01, 0.07]	0.1105
Group x Time x INR				
Pre-pandemic x Year 1	0.00	0.00		
Pre-pandemic x Year 2	0.00	0.00		
Intra-pandemic x Year 1	0.00	0.00		
Intra-pandemic x Year 2	0.23	0.07	[0.09, 0.37]	0.0016

Note. GED = Generalized Education Diploma; INR = income-to-needs ratio.

© 2024 Adise S et al. *JAMA Network Open.*

eTable10: The estimated regression coefficient with 95% CI from mixed-effects regression analysis of CBCL Withdrawn/Depressed raw score between pre- and intra-pandemic groups modified by INR, adjusting for inter-study interval, education, age, and sex.

Variable	Coef.	SE	[95% CI]	p-value
Age	0.01	0.00	[0.01, 0.02]	0.0000
Sex				
Female	0.00	0.00		
Male	-0.01	0.03	[-0.07, 0.06]	0.8597
Education				
< High school (HS) Diploma	0.00	0.00		
HS Diploma/GED	-0.14	0.10	[-0.34, 0.07]	0.1894
Some College	-0.02	0.09	[-0.20, 0.16]	0.8291
Bachelor's Degree	-0.16	0.09	[-0.34, 0.03]	0.0982
Postgraduate Degree	-0.20	0.10	[-0.39, -0.01]	0.0416
Inter-study interval (months)	0.01	0.01	[-0.01, 0.03]	0.3581
INR	-0.06	0.01	[-0.08, -0.04]	0.0000
Group				
Pre-pandemic	0.00	0.00		
Intra-pandemic	-0.01	0.09	[-0.17, 0.16]	0.9537
Time				
Year 1	0.00	0.00		
Year 2	-0.07	0.04	[-0.16, 0.02]	0.1262
Group x Time				
Pre-pandemic x Year 1	0.00	0.00		
Pre-pandemic x Year 2	0.00	0.00		
Intra-pandemic x Year 1	0.00	0.00		
Intra-pandemic x Year 2	-0.06	0.13	[-0.31, 0.20]	0.6613
Group x INR				
Pre-pandemic	0.00	0.00		
Intra-pandemic	-0.01	0.02	[-0.04, 0.03]	0.6816
Time x INR				
Year 1	0.00	0.00		
Year 2	0.01	0.01	[-0.01, 0.02]	0.3833
Group x Time x INR				
Pre-pandemic x Year 1	0.00	0.00		
Pre-pandemic x Year 2	0.00	0.00		
Intra-pandemic x Year 1	0.00	0.00		
Intra-pandemic x Year 2	0.03	0.03	[-0.02, 0.09]	0.2648

Note. GED = Generalized Education Diploma; INR = income-to-needs ratio.

© 2024 Adise S et al. *JAMA Network Open.*

eTable11: The estimated regression coefficient with 95% CI from mixed-effects regression analysis of CBCL Somatic Complaints raw score between pre- and intra-pandemic groups modified by INR, adjusting for inter-study interval, education, age, and sex.

Variable	Coef.	SE	[95% CI]	p-value
Age	0.00	0.00	[-0.00, 0.01]	0.3691
Sex				
Female	0.00	0.00		
Male	-0.16	0.04	[-0.23, -0.09]	0.0000
Education				
< High school (HS) Diploma	0.00	0.00		
HS Diploma/GED	-0.04	0.11	[-0.25, 0.18]	0.7343
Some College	0.21	0.10	[0.03, 0.40]	0.0247
Bachelor's Degree	0.10	0.10	[-0.09, 0.30]	0.3084
Postgraduate Degree	0.01	0.10	[-0.20, 0.21]	0.9506
Inter-study interval (months)	0.02	0.01	[0.00, 0.04]	0.0267
INR	-0.05	0.01	[-0.07, -0.03]	0.0000
Group				
Pre-pandemic	0.00	0.00		
Intra-pandemic	-0.16	0.09	[-0.34, 0.02]	0.0862
Time				
Year 1	0.00	0.00		
Year 2	-0.08	0.05	[-0.17, 0.02]	0.1198
Group x Time				
Pre-pandemic x Year 1	0.00	0.00		
Pre-pandemic x Year 2	0.00	0.00		
Intra-pandemic x Year 1	0.00	0.00		
Intra-pandemic x Year 2	-0.24	0.14	[-0.53, 0.04]	0.0879
Group x INR				
Pre-pandemic	0.00	0.00		
Intra-pandemic	0.02	0.02	[-0.01, 0.06]	0.1876
Time x INR				
Year 1	0.00	0.00		
Year 2	0.00	0.01	[-0.01, 0.02]	0.7330
Group x Time x INR				
Pre-pandemic x Year 1	0.00	0.00		
Pre-pandemic x Year 2	0.00	0.00		
Intra-pandemic x Year 1	0.00	0.00		
Intra-pandemic x Year 2	0.05	0.03	[-0.02, 0.11]	0.1433

Note. GED = Generalized Education Diploma; INR = income-to-needs ratio.

© 2024 Adise S et al. *JAMA Network Open.*

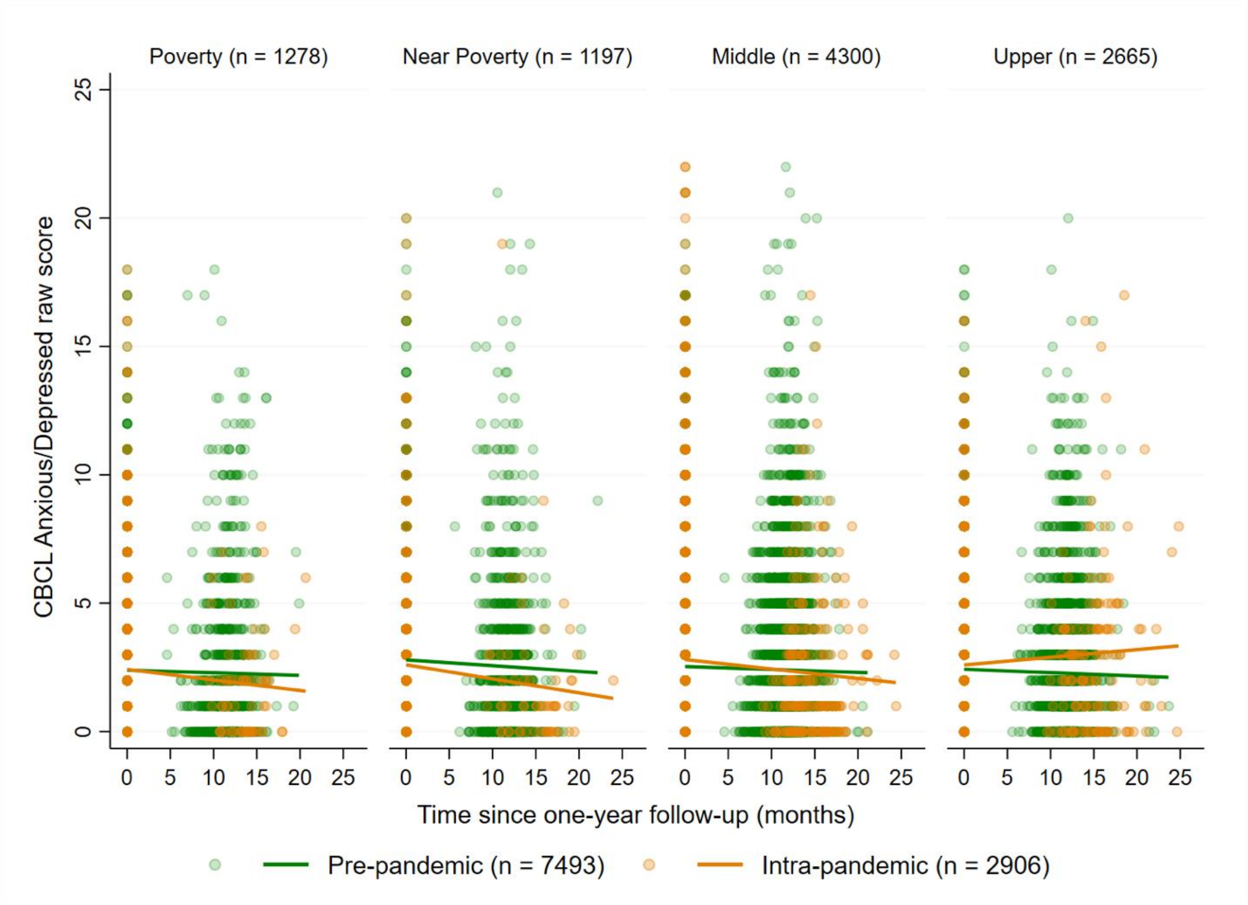

eFigure1. Each panel shows associations between CBCL Anxious/Depressed score at 1-year and 2-year follow-ups by different income-to-needs ratio (INR) category (poverty: < 100%; near poverty: 100% - < 200%; middle: 200% - < 600%; upper: ≥ 600%). The observed data points (circles) and best-fitted regression lines are shown in green and orange for pre-pandemic and intra-pandemic groups, respectively. The figure illustrates a decrease over time in Anxious/Depressed symptoms among youth from families experiencing poverty and near-poverty, who were also exposed to the pandemic, compared to those in the pre-pandemic group. Conversely, it indicates elevated symptoms over time among youth from upper-income families exposed to the pandemic. Although the symptoms among youth in the intra-pandemic group from middle-income families appear to have slightly decreased, they remain nearly similar to that of their counterparts in the pre-pandemic group.

© 2024 Adise S et al. *JAMA Network Open.*

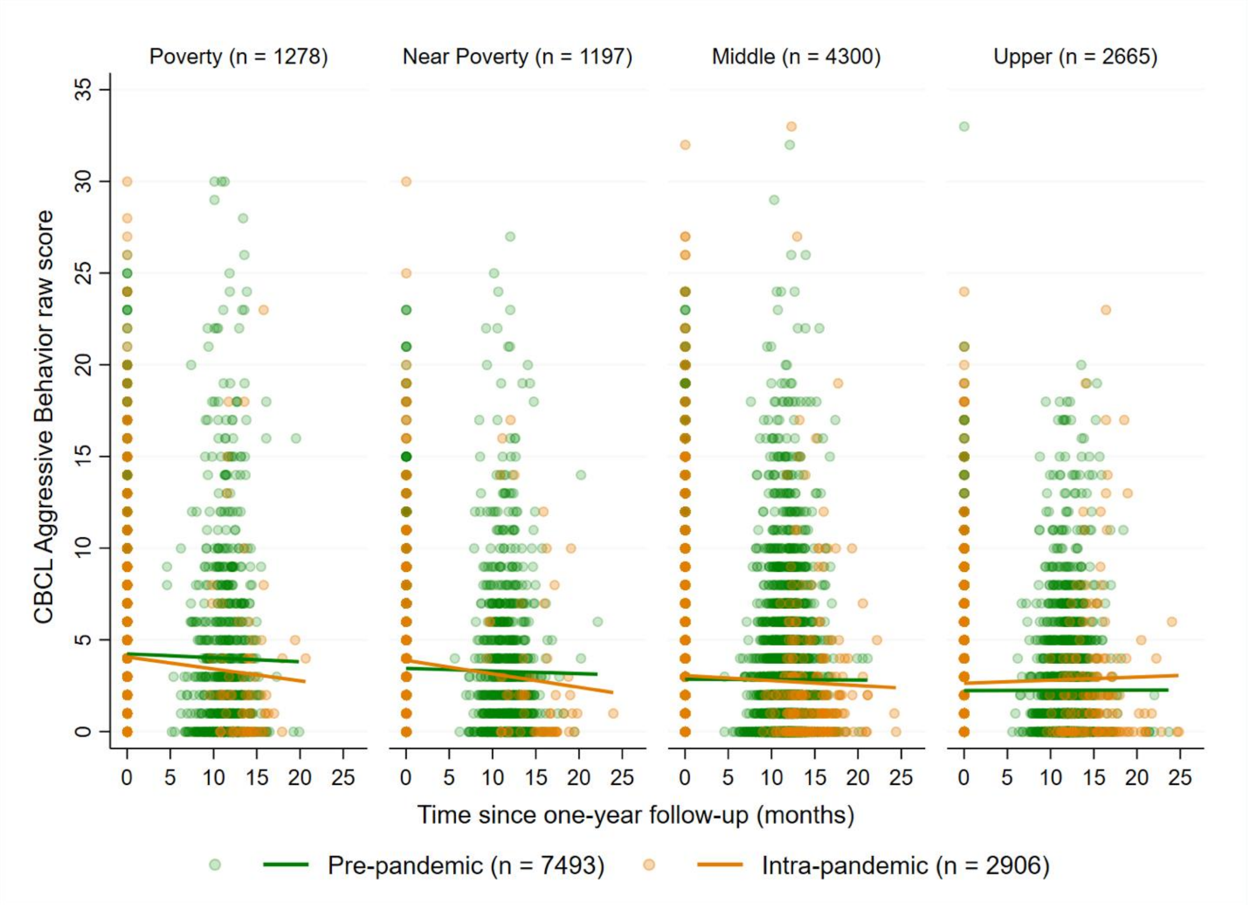

eFigure2. Each panel shows associations between CBCL Aggressive Behavior score at 1-year and 2-year follow-ups by different income-to-needs ratio (INR) category (poverty: < 100%; near poverty: 100% - < 200%; middle: 200% - < 600%; upper: ≥ 600%). The observed data points (circles) and best-fitted regression lines are shown in green and orange for pre-pandemic and intra-pandemic groups, respectively. Notably, the figure indicates reduced behavioral issues among individuals exposed to pandemic in the poverty category, and conversely, elevated problems in upper-income category. The figure illustrates a decrease over time in Aggressive Behavior among youth from families experiencing poverty and near-poverty, who were also exposed to the pandemic, compared to those in the pre-pandemic group. Conversely, it indicates an increase in Aggressive Behavior over time among youth from upper-income families exposed to the pandemic. Although Aggressive Behavior among youth in the intra-pandemic group from middle-income families appear to have slightly decreased, it remains almost identical to that of their counterparts in the pre-pandemic group.

© 2024 Adise S et al. *JAMA Network Open.*

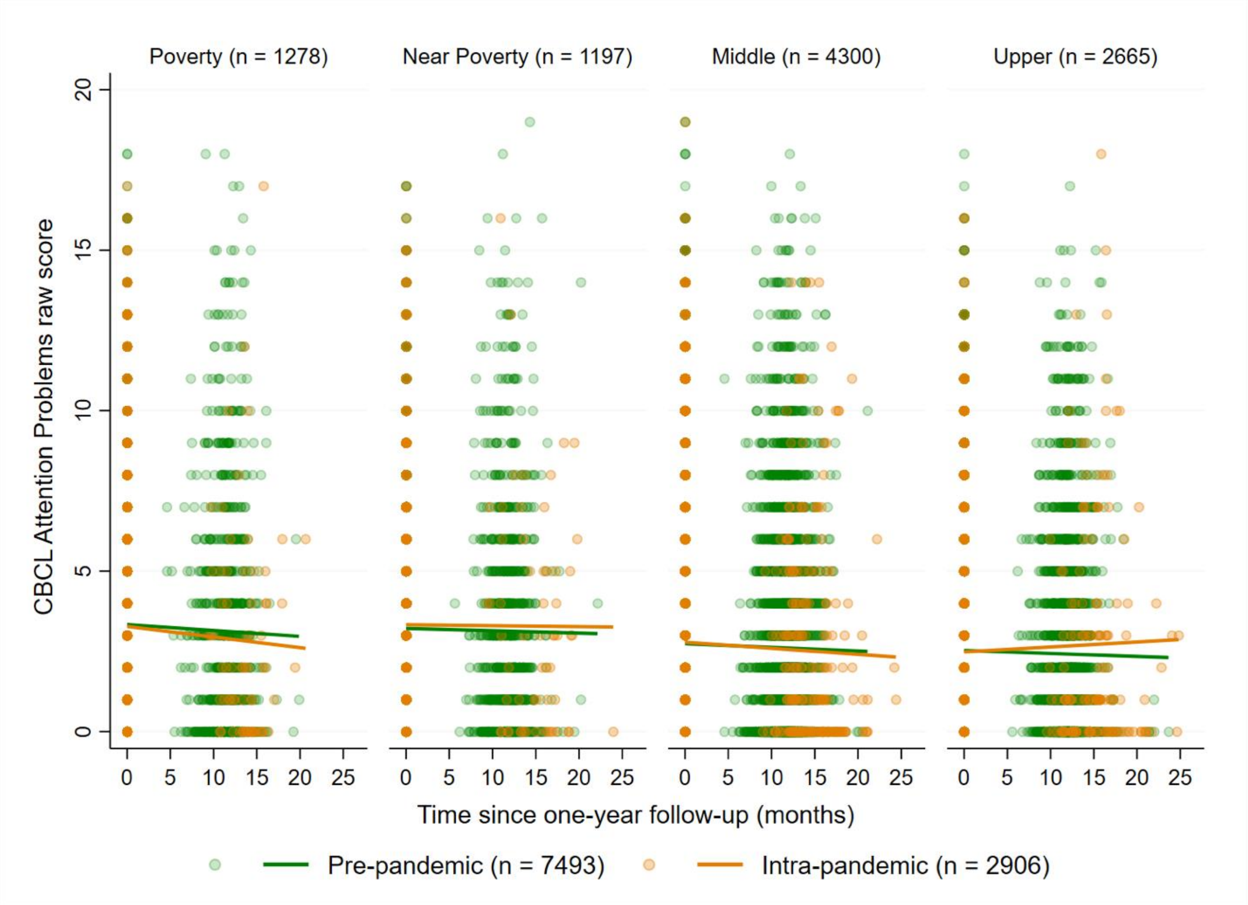

eFigure3. Each panel shows associations between CBCL Attention Problems score at 1-year and 2-year follow-ups by different income-to-needs ratio (INR) category (poverty: < 100%; near poverty: 100% - < 200%; middle: 200% - < 600%; upper: ≥ 600%). The observed data points (circles) and best-fitted regression lines are shown in green and orange for pre-pandemic and intra-pandemic groups, respectively. The figure illustrates a decrease in Attention Problems over time among youth from families experiencing poverty and pandemic exposure compared to those in the pre-pandemic group. Conversely, it indicates an increase in problems over time among youth from upper-income families exposed to the pandemic. While Attention Problems have stayed relatively stable among youth in the intra-pandemic group from near-poverty families, a slight decrease is observed in this group from middle-income families, with levels nearly identical to those in the pre-pandemic group.

© 2024 Adise S et al. *JAMA Network Open.*

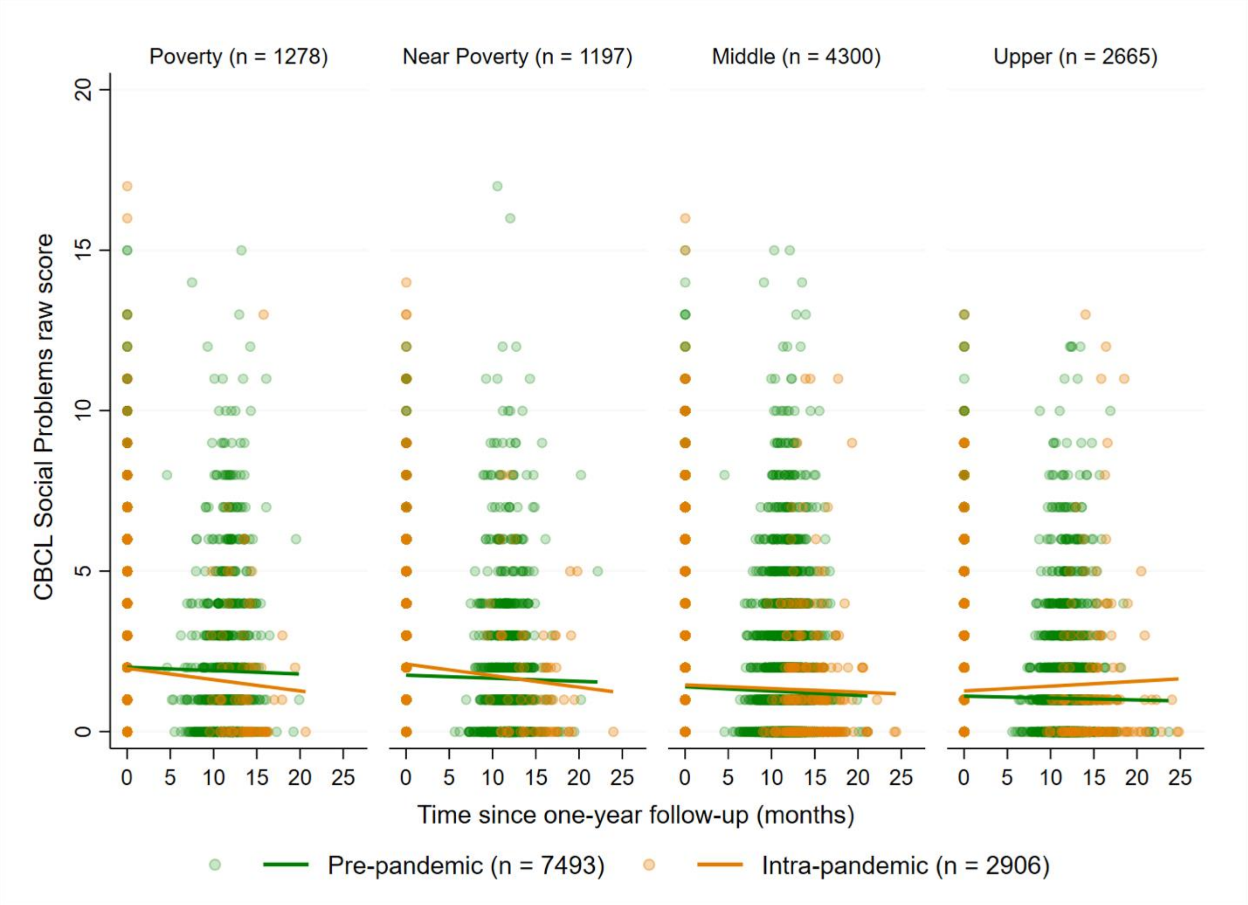

eFigure4. Each panel shows associations between CBCL Social Problems score at 1-year and 2-year follow-ups by different income-to-needs ratio (INR) category (poverty: < 100%; near poverty: 100% - < 200%; middle: 200% - < 600%; upper: ≥ 600%). The observed data points (circles) and best-fitted regression lines are shown in green and orange for pre-pandemic and intra-pandemic groups, respectively. The figure illustrates a decrease over time in Social Problems among youth from families experiencing poverty and near-poverty, who were also exposed to the pandemic, compared to those in the pre-pandemic group. Conversely, it indicates elevated problems over time among youth from upper-income families exposed to the pandemic. Notably, Social Problems have shown a slight decrease among youth in the intra-pandemic group from middle-income families, with levels almost similar to those in the pre-pandemic group.

© 2024 Adise S et al. *JAMA Network Open.*

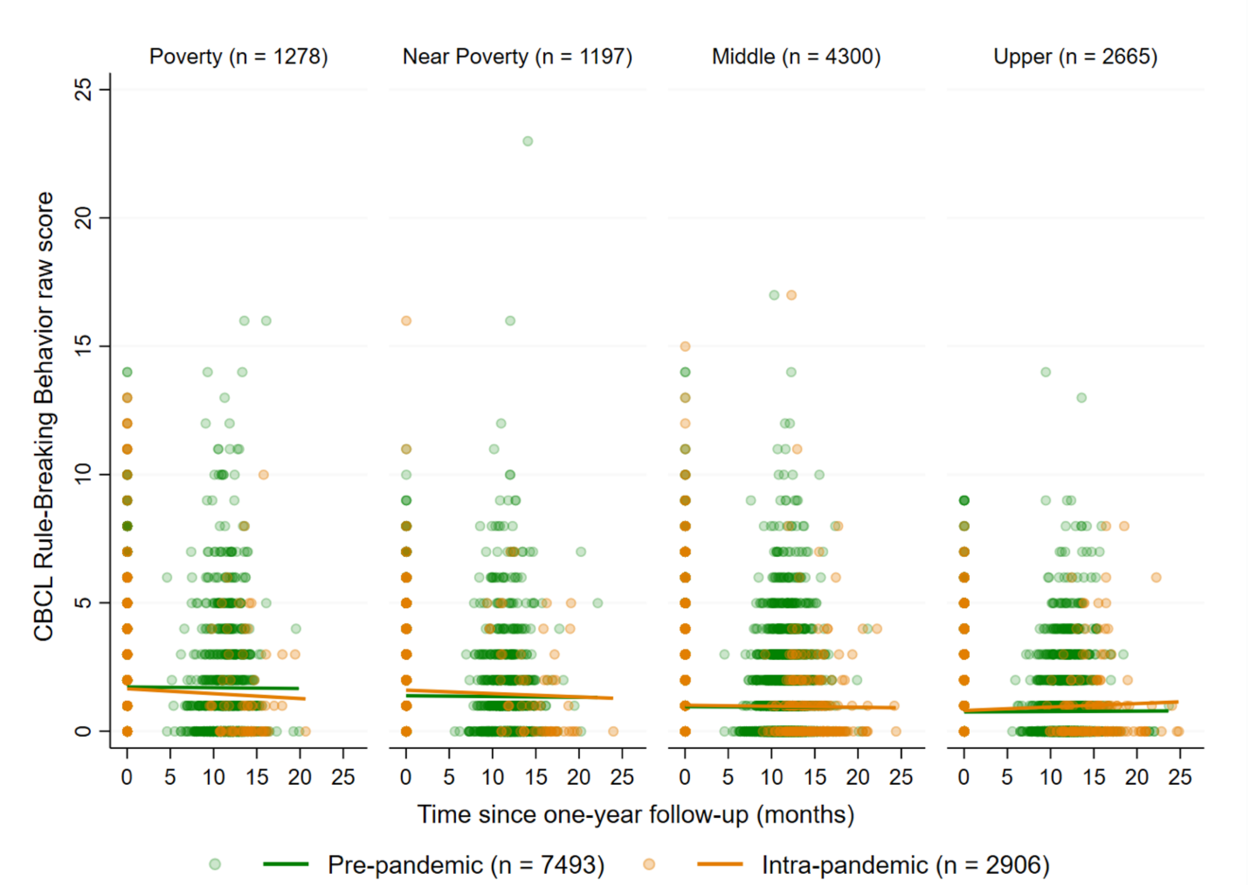

eFigure5. Each panel shows associations between CBCL Rule-Breaking Behavior score at 1-year and 2-year follow-ups by different income-to-needs ratio (INR) category (poverty: < 100%; near poverty: 100% - < 200%; middle: 200% - < 600%; upper: ≥ 600%). The observed data points (circles) and best-fitted regression lines are shown in green and orange for pre-pandemic and intra-pandemic groups, respectively. The figure illustrates a slight decrease over time in Rule-Breaking Behavior among youth from families experiencing poverty and pandemic exposure compared to those in the pre-pandemic group. Conversely, it indicates a slight increase over time in this behavior among youth from upper-income families exposed to the pandemic. Notably, Rule-Breaking Behavior has remained unchanged among youth in the intra-pandemic group from near-poverty and middle-income families, with levels almost identical to those in the pre-pandemic group.

© 2024 Adise S et al. *JAMA Network Open.*

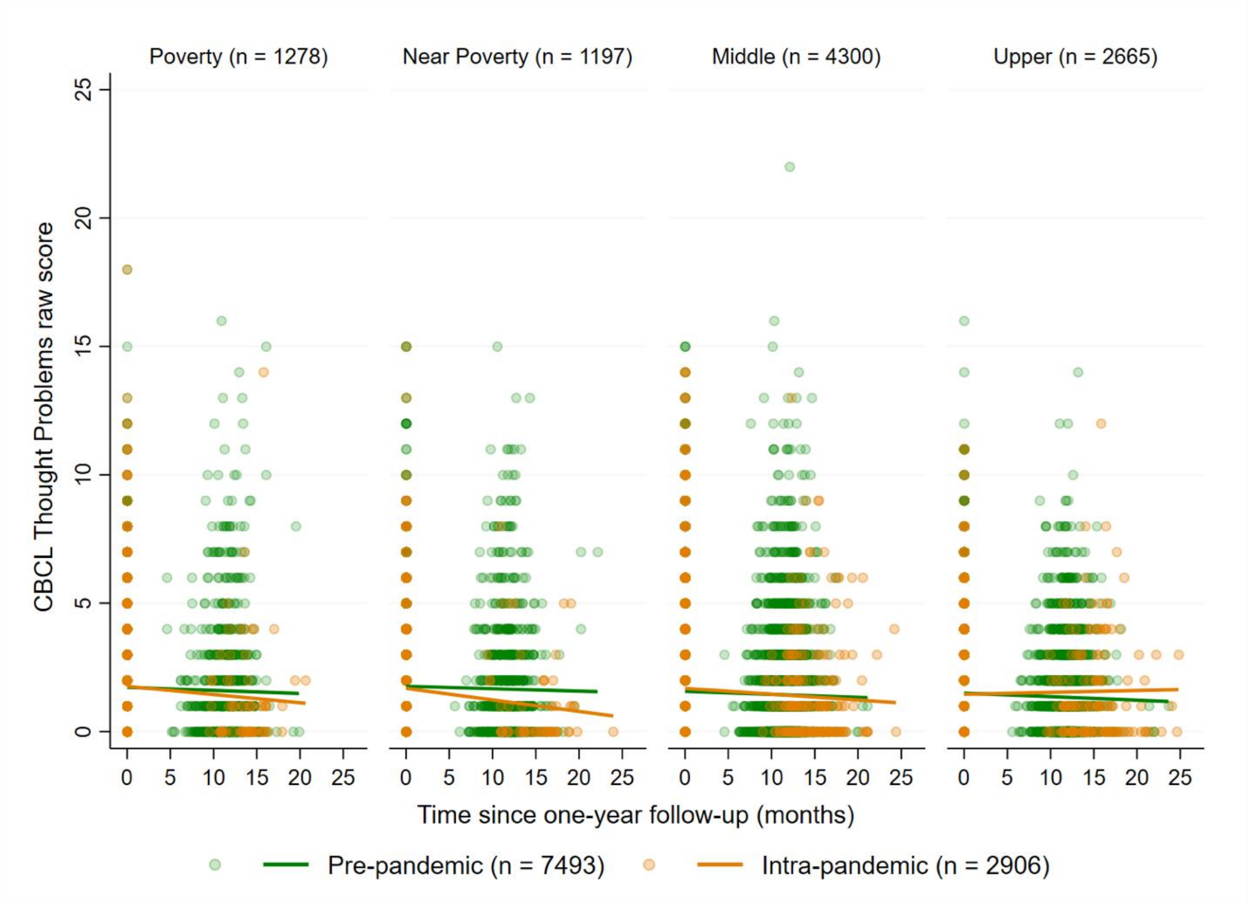

eFigure6. Each panel shows associations between CBCL Thought Problems score at 1-year and 2-year follow-ups by different income-to-needs ratio (INR) category (poverty: < 100%; near poverty: 100% - < 200%; middle: 200% - < 600%; upper: ≥ 600%). The observed data points (circles) and best-fitted regression lines are shown in green and orange for pre-pandemic and intra-pandemic groups, respectively. The figure illustrates a decrease over time in Thought Problems among youth from families experiencing poverty and near-poverty, who were also exposed to the pandemic, compared to those in the pre-pandemic group. Conversely, it indicates elevated problems over time among youth from upper-income families exposed to the pandemic. Notably, Thought Problems have almost remained constant among youth in intra-pandemic group from middle-income families, with levels similar to those in the pre-pandemic group.

© 2024 Adise S et al. *JAMA Network Open.*

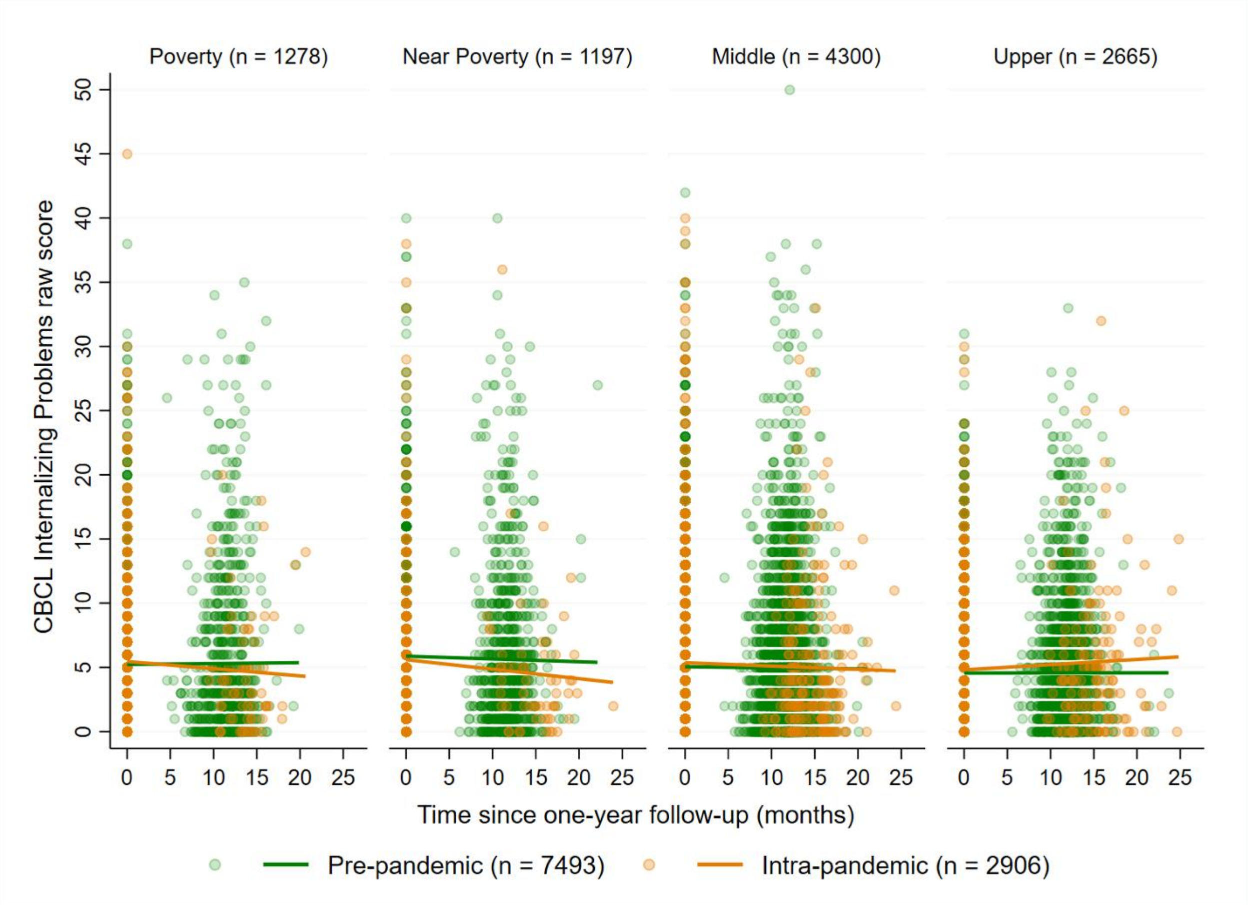

eFigure7. Each panel shows associations between CBCL Internalizing Problems score at 1-year and 2-year follow-ups by different income-to-needs ratio (INR) category (poverty: < 100%; near poverty: 100% - < 200%; middle: 200% - < 600%; upper: ≥ 600%). The observed data points (circles) and best-fitted regression lines are shown in green and orange for pre-pandemic and intra-pandemic groups, respectively. The figure illustrates a decrease over time in Internalizing Problems among youth from families experiencing poverty and near-poverty, who were also exposed to the pandemic, compared to those in the pre-pandemic group. Conversely, it indicates an increase in Internalizing Problems over time among youth from upper-income families exposed to the pandemic. Notably, Internalizing Problems remain relatively stable among youth in the intra-pandemic group from middle-income families, showing levels almost identical to that of their counterparts in the pre-pandemic group.

© 2024 Adise S et al. *JAMA Network Open.*

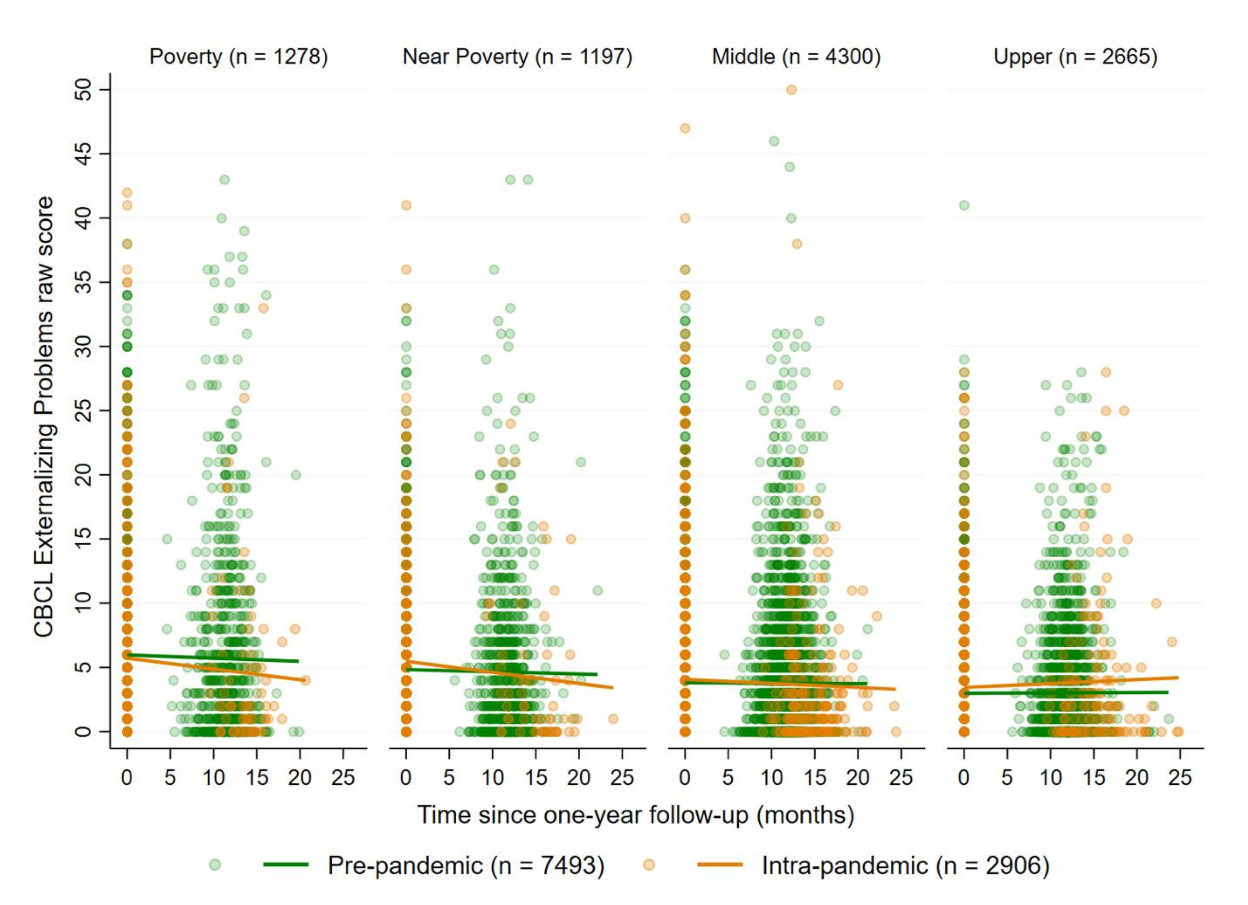

eFigure8. Each panel shows associations between CBCL Externalizing Problems score at 1-year and 2-year follow-ups by different income-to-needs ratio (INR) category (poverty: < 100%; near poverty: 100% - < 200%; middle: 200% - < 600%; upper: ≥ 600%). The observed data points (circles) and best-fitted regression lines are shown in green and orange for pre-pandemic and intra-pandemic groups, respectively. The figure illustrates a decrease over time in Externalizing Problems among youth from families experiencing poverty and near-poverty, who were also exposed to the pandemic, compared to those in the pre-pandemic group. Conversely, it indicates an increase in Externalizing Problems over time among youth from upper-income families exposed to the pandemic. Notably, Externalizing Problems remain relatively stable among youth in the intra-pandemic group from middle-income families, exhibiting levels almost identical to that of their counterparts in the pre-pandemic group.

© 2024 Adise S et al. *JAMA Network Open.*

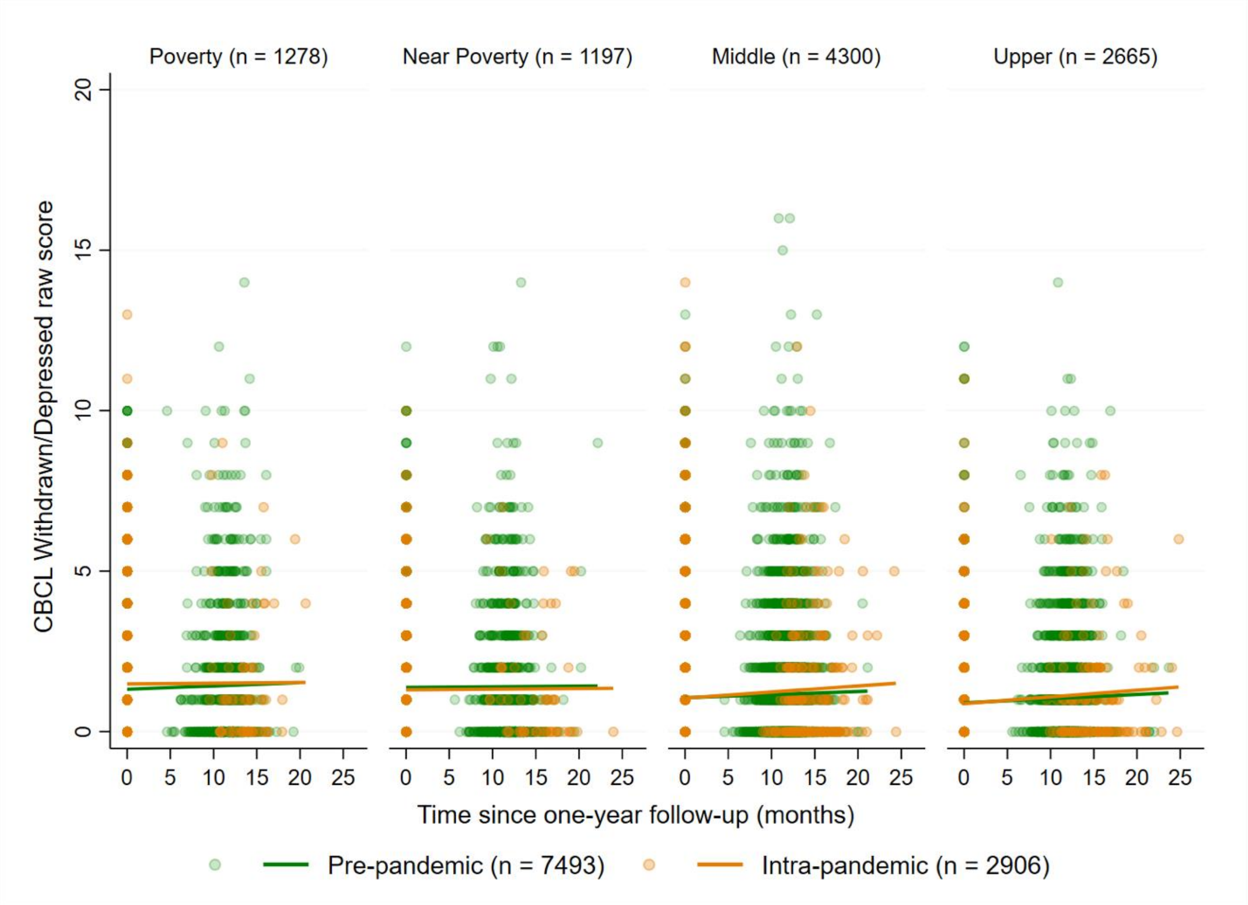

eFigure9. Each panel shows associations between CBCL Withdrawn/Depressed score at 1-year and 2-year follow-ups by different income-to-needs ratio (INR) category (poverty: < 100%; near poverty: 100% - < 200%; middle: 200% - < 600%; upper: ≥ 600%). The observed data points (circles) and best-fitted regression lines are shown in green and orange for pre-pandemic and intra-pandemic groups, respectively. The figure illustrates Withdrawn/Depressed symptoms remaining relatively stable over time among youth in the intra-pandemic group across different INR categories, with levels almost identical to those of their counterparts in the pre-pandemic group.

© 2024 Adise S et al. *JAMA Network Open.*

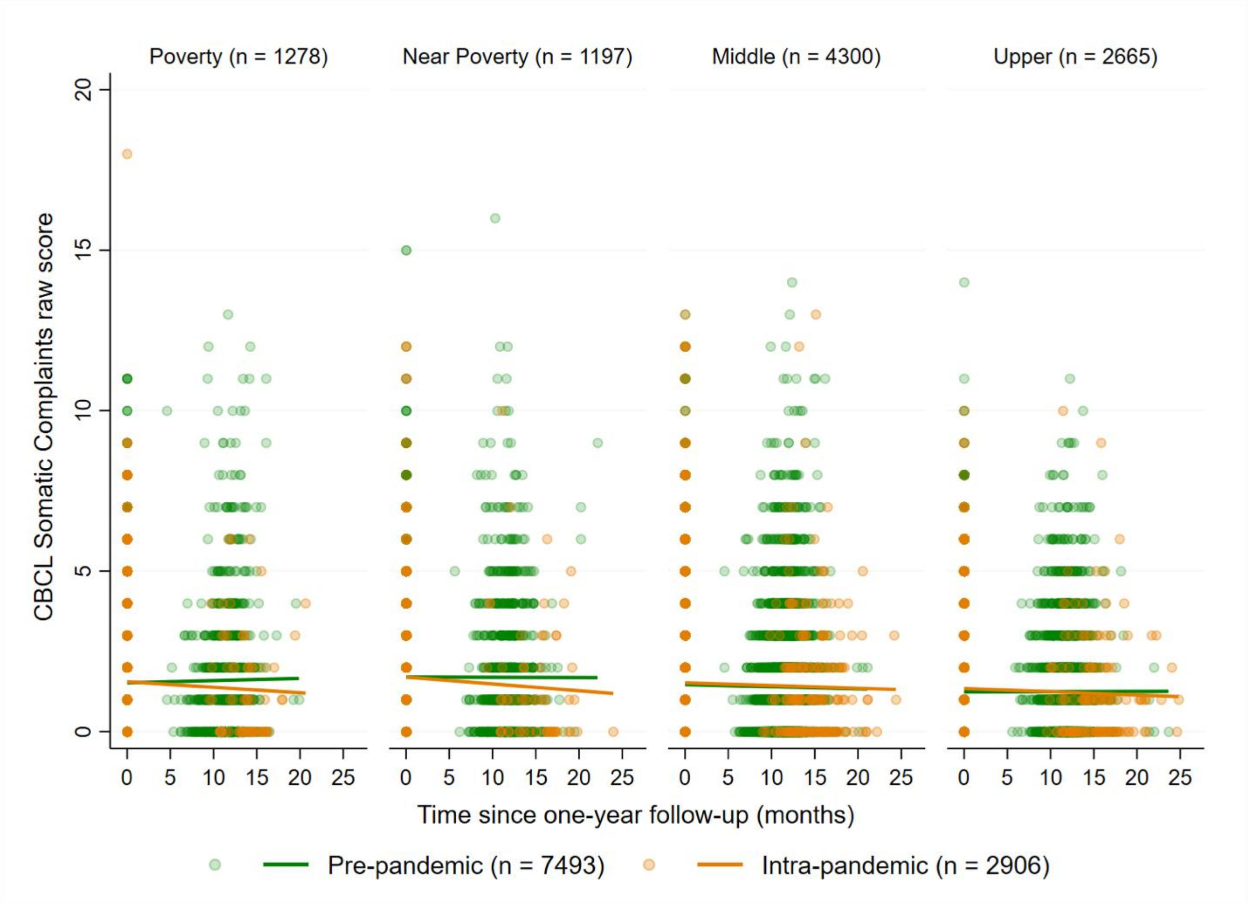

eFigure10. Each panel shows associations between CBCL Somatic Complaints score at 1-year and 2-year follow-ups by different income-to-needs ratio (INR) category (poverty: < 100%; near poverty: 100% - < 200%; middle: 200% - < 600%; upper: ≥ 600%). The observed data points (circles) and best-fitted regression lines are shown in green and orange for pre-pandemic and intra-pandemic groups, respectively. The figure illustrates a decrease over time in Somatic Complaints among youth from families experiencing poverty and near-poverty, who were also exposed to the pandemic, compared to those in the pre-pandemic group. Notably, Somatic Complaints have remained constant among youth in the intra-pandemic group from middle-income and upper income families, with levels almost identical to those of their counterparts in the pre-pandemic group.

© 2024 Adise S et al. *JAMA Network Open.*
