# Supplementary material for: Socioeconomic Disadvantage and Youth Mental Health During the COVID-19 Pandemic Lockdown
Source: JAMA Netw Open. 2024 Jul 5;7(7):e2420466. doi: 10.1001/jamanetworkopen.2024.20466 (PMC11227076; doi:10.1001/jamanetworkopen.2024.20466)
Supplement: Supplement 2. — Data Sharing Statement [file jamanetwopen-e2420466-s002.pdf]

Data Sharing Statement

Adise. Socioeconomic Disadvantage and Youth Mental Health During the COVID-19 Pandemic Lockdown. *JAMA Netw Open*. Published July 05, 2024.
doi:10.1001/jamanetworkopen.2024.20466

Data
Data available: No

Additional Information
Explanation for why data not available: these data were gathered from the abcd study which others can get access to via approval from the national data archives.
